# Runners in Action

**DOI:** 10.1007/978-3-030-44914-8_2

**Published:** 2020-04-18

**Authors:** Danel Ahman, Andrej Bauer

**Affiliations:** ETH Zurich, Zurich, Switzerland; Faculty of Mathematics and Physics, University of Ljubljana, Ljubljana, Slovenia

**Keywords:** Runners, comodels, algebraic effects, resources, finalisation.

## Abstract

Runners of algebraic effects, also known as comodels, provide a mathematical model of resource management. We show that they also give rise to a programming concept that models top-level external resources, as well as allows programmers to modularly define their own intermediate “virtual machines”. We capture the core ideas of programming with runners in an equational calculus $\lambda _{\mathsf {coop}}$, which we equip with a sound and coherent denotational semantics that guarantees the linear use of resources and execution of finalisation code. We accompany $\lambda _{\mathsf {coop}}$ with examples of runners in action, provide a prototype language implementation in OCaml, as well as a Haskell library based on $\lambda _{\mathsf {coop}}$.

### 1 Introduction

Computational effects, such as exceptions, input-output, state, nondeterminism, and randomness, are an important component of general-purpose programming languages, whether they adopt functional, imperative, object-oriented, or other programming paradigms. Even pure languages exhibit computational effects at the top level, so to speak, by interacting with their external environment.

In modern languages, computational effects are often structured using *monads* [22,23,36], or *algebraic effects and handlers* [12,28,30]. These mechanisms excel at implementation of computational effects within the language itself. For instance, the familiar implementation of mutable state in terms of state-passing functions requires no native state, and can be implemented either as a monad or using handlers. One is naturally drawn to using these techniques also for dealing with actual effects, such as manipulation of native memory and access to hardware. These are represented inside the language as algebraic operations (as in Eff [4]) or a monad (in the style of Haskell's IO), but treated specially by the language's top-level runtime, which invokes corresponding operating system functionality. While this approach works in practice, it has some unfortunate downsides too, namely *lack of modularity and linearity*, and *excessive generality*.

Lack of modularity is caused by having the external resources hard-coded into the top-level runtime. As a result, changing which resources are available and how they are implemented requires modifications of the language implementation. Additional complications arise when a language supports several operating systems and hardware platforms, each providing their own, different feature set.

One wishes that the ingenuity of the language implementors were better supported by a more flexible methodology with a sound theoretical footing.

Excessive generality is not as easily discerned, because generality of programming concepts makes a language expressive and useful, such as general algebraic effects and handlers enabling one to implement timeouts, rollbacks, stream redirection [30], async & await [16], and concurrency [9]. However, the flip side of such expressive freedom is the lack of any guarantees about how external resources will actually be used. For instance, consider a simple piece of code, written in EFF-like syntax, which first opens a file, then writes to it, and finally closes it:

```
let fh = open "hello.txt" in write (fh, "Hello, world."); close fh
```

What this program actually does depends on how the operations open, write, and close are handled. For all we know, an enveloping handler may intercept the write operation and discard its continuation, so that close never happens and the file is not properly closed. Telling the programmer not to shoot themselves in the foot by avoiding such handlers is not helpful, because the handler may encounter an external reason for not being able to continue, say a full disk.

Even worse, external resources may be misused accidentally when we combine two handlers, each of which works as intended on its own. For example, if we combine the above code with a non-deterministic choose operation, as in

```
let fh = open "greeting.txt" in
let b = choose () in
if b then write (fh, "hello") else write (fh, "good bye") ; close fh
```

and handle it with the standard non-determinism handler

```
handler { return x → [x], choose () k → return (append (k true) (k false)) }
```

The resulting program attempts to close the file twice, as well as write to it twice, because the continuation k is invoked twice when handling choose. Of course, with enough care all such situations can be dealt with, but that is beside the point. It is worth sacrificing some amount of the generality of algebraic effects and monads in exchange for predictable and safe usage of external computational effects, so long as the vast majority of common use cases are accommodated.

**Contributions** We address the described issues by showing how to design a programming language based on *runners of algebraic effects*. We review runners in §2 and recast them as a programming construct in §3. In §4, we present $\lambda_{\mathsf{coop}}$, a calculus that captures the core ideas of programming with runners. We provide a coherent and sound denotational semantics for $\lambda_{\mathsf{coop}}$ in §5, where we also prove that well-typed code is properly finalised. In §6, we show examples of runners in action. The paper is accompanied by a prototype language COOP and a HASKELL library HASKELL-COOP, based on $\lambda_{\mathsf{coop}}$, see §7. The relationship between $\lambda_{\mathsf{coop}}$ and existing work is addressed in §8, and future possibilities discussed in §9.

The paper is also accompanied by an online appendix (https://arxiv.org/abs/1910.11629) that provides the typing and equational rules we omit in §4.

Runners are *modular* in that they can be used not only to model the top-level interaction with the external environment, but programmers can also use them to define and nest their own intermediate "virtual machines". Our runners are *effectful*: they may handle operations by calling further outer operations, and raise exceptions and send signals, through which exceptional conditions and runtime errors are communicated back to user programs in a safe fashion that preserves linear usage of external resources and ensures their proper finalisation.

We achieve *suitable generality* for handling of external resources by showing how runners provide implementations of algebraic operations together with a natural notion of finalisation, and a strong guarantee that in the absence of external kill signals the finalisation code is executed exactly once (Thm. 7). We argue that for most purposes such discipline is well worth having, and giving up the arbitrariness of effect handlers is an acceptable price to pay. In fact, as will be apparent in the denotational semantics, runners are simply a restricted form of handlers, which apply the continuation at most once in a tail call position.

Runners guarantee *linear usage of resources* not through a linear or uniqueness type system (such as in the CLEAN programming language [15]) or a syntactic discipline governing the application of continuations in handlers, but rather by a design based on the linear state-passing technique studied by Møgelberg and Staton [21]. In this approach, a computational resource may be implemented without restrictions, but is then guaranteed to be used linearly by user code.

### 2 Algebraic effects, handlers, and runners

We begin with a short overview of the theory of algebraic effects and handlers, as well as runners. To keep focus on how runners give rise to a programming concept, we work naively in set theory. Nevertheless, we use category-theoretic language as appropriate, to make it clear that there are no essential obstacles to extending our work to other settings (we return to this point in §5.1).

#### 2.1 Algebraic effects and handlers

There is by now no lack of material on the algebraic approach to structuring computational effects. For an introductory treatment we refer to [5], while of course also recommend the seminal papers by Plotkin and Power [25,28]. The brief summary given here only recalls the essentials and introduces notation.

An *(algebraic) signature* is given by a set $\Sigma$ of *operation symbols*, and for each $\mathsf{op} \in \Sigma$ its *operation signature* $\mathsf{op} : A_{\mathsf{op}} \leadsto B_{\mathsf{op}}$, where $A_{\mathsf{op}}$ and $B_{\mathsf{op}}$ are called the *parameter* and *arity* set. A *$\Sigma$-structure* $\mathcal{M}$ is given by a carrier set $|\mathcal{M}|$, and for each operation symbol $\mathsf{op} \in \Sigma$, a map $\mathsf{op}_{\mathcal{M}} : A_{\mathsf{op}} \times (B_{\mathsf{op}} \Rightarrow |\mathcal{M}|) \to |\mathcal{M}|$, where $\Rightarrow$ is set exponentiation. The *free $\Sigma$-structure* $\mathrm{Tree}_{\Sigma}(X)$ over a set $X$ is the set of well-founded trees generated inductively by

- $\mathsf{return}\, x \in \mathrm{Tree}_{\Sigma}(X)$, for every $x \in X$, and
- $\mathsf{op}(a, \kappa) \in \mathrm{Tree}_{\Sigma}(X)$, for every $\mathsf{op} \in \Sigma$, $a \in A_{\mathsf{op}}$, and $\kappa : B_{\mathsf{op}} \to \mathrm{Tree}_{\Sigma}(X)$.

We are abusing notation in a slight but standard way, by using $\mathsf{op}$ both as the name of an operation and a tree-forming constructor. The elements of $\mathrm{Tree}_{\Sigma}(X)$ are called *computation trees*: a leaf $\mathsf{return}\, x$ represents a pure computation returning a value $x$, while $\mathsf{op}(a, \kappa)$ represents an effectful computation that calls $\mathsf{op}$ with parameter $a$ and continuation $\kappa$, which expects a result from $B_{\mathsf{op}}$.

An *algebraic theory* $\mathcal{T} = (\Sigma_{\mathcal{T}}, \mathrm{Eq}_{\mathcal{T}})$ is given by a *signature* $\Sigma_{\mathcal{T}}$ and a set of *equations* $\mathrm{Eq}_{\mathcal{T}}$. The equations $\mathrm{Eq}_{\mathcal{T}}$ express computational behaviour via interactions between operations, and are written in a suitable formalism, e.g., [30]. We explain these by way of examples, as the precise details do not matter for our purposes. Let $\mathbb{0} = \{\,\}$ be the empty set and $\mathbb{1} = \{\star\}$ the standard singleton.

*Example 1.* Given a set $C$ of possible states, the theory of *$C$-valued state* has two operations, whose somewhat unusual naming will become clear later on,

$$\mathsf{getenv} : \mathbb{1} \rightsquigarrow C, \qquad \mathsf{setenv} : C \rightsquigarrow \mathbb{1}$$

and the equations (where we elide appearances of $\star$):

$$\mathsf{getenv}(\lambda c \,.\, \mathsf{setenv}(c, \kappa)) = \kappa, \qquad \mathsf{setenv}(c, \mathsf{getenv}\, \kappa) = \mathsf{setenv}(c, \kappa\, c),$$
$$\mathsf{setenv}(c, \mathsf{setenv}(c', \kappa)) = \mathsf{setenv}(c', \kappa).$$

For example, the second equation states that reading state right after setting it to $c$ gives precisely $c$. The third equation states that $\mathsf{setenv}$ overwrites the state.

*Example 2.* Given a set of exceptions $E$, the algebraic theory of *$E$-many exceptions* is given by a single operation $\mathsf{raise} : E \rightsquigarrow \mathbb{0}$, and no equations.

A *$\mathcal{T}$-model*, also called a *$\mathcal{T}$-algebra*, is a $\Sigma_{\mathcal{T}}$-structure which satisfies the equations in $\mathrm{Eq}_{\mathcal{T}}$. The *free $\mathcal{T}$-model* over a set $X$ is constructed as the quotient

$$\mathrm{Free}_{\mathcal{T}}(X) = \mathrm{Tree}_{\Sigma_{\mathcal{T}}}(X)/{\sim}$$

by the $\Sigma_{\mathcal{T}}$-congruence $\sim$ generated by $\mathrm{Eq}_{\mathcal{T}}$. Each $\mathsf{op} \in \Sigma_{\mathcal{T}}$ is interpreted in the free model as the map $(a, \kappa) \mapsto [\mathsf{op}(a, \kappa)]$, where $[-]$ is the $\sim$-equivalence class.

$\mathrm{Free}_{\mathcal{T}}(-)$ is the functor part of a *monad* on sets, whose *unit* at a set $X$ is

$$X \xrightarrow{\;\mathsf{return}\;} \mathrm{Tree}_{\Sigma_{\mathcal{T}}}(X) \xrightarrow{\;[-]\;} \mathrm{Free}_{\mathcal{T}}(X)\,.$$

The *Kleisli extension* for this monad is then the operation which lifts any map $f : X \to \mathrm{Tree}_{\Sigma_{\mathcal{T}}}(Y)$ to the map $f^{\dagger} : \mathrm{Free}_{\Sigma_{\mathcal{T}}}(X) \to \mathrm{Free}_{\Sigma_{\mathcal{T}}}(Y)$, given by

$$f^{\dagger}\,[\mathsf{return}\, x] \stackrel{\text{def}}{=} f\, x, \qquad f^{\dagger}\,[\mathsf{op}(a, \kappa)] \stackrel{\text{def}}{=} [\mathsf{op}(a, f^{\dagger} \circ \kappa)].$$

That is, $f^{\dagger}$ traverses a computation tree and replaces each leaf $\mathsf{return}\, x$ with $f\, x$.

The preceding construction of free models and the monad may be retrofitted to an algebraic signature $\Sigma$, if we construe $\Sigma$ as an algebraic theory with no equations. In this case $\sim$ is just equality, and so we may omit the quotient and the pesky equivalence classes. Thus the carrier of the free $\Sigma$-model is the set of well-founded trees $\mathrm{Tree}_{\Sigma}(X)$, with the evident monad structure.

A fundamental insight of Plotkin and Power [25,28] was that many computational effects may be adequately described by algebraic theories, with the elements of free models corresponding to effectful computations. For example, the monads induced by the theories from Examples 1 and 2 are respectively isomorphic to the usual *state monad* $\mathsf{St}_C\, X \stackrel{\text{def}}{=} (C \Rightarrow X \times C)$ and the *exceptions monad* $\mathsf{Exc}_E\, X \stackrel{\text{def}}{=} X + E$.

Plotkin and Pretnar [30] further observed that the universal property of free models may be used to model a programming concept known as *handlers*. Given a $\mathcal{T}$-model $\mathcal{M}$ and a map $f : X \to |\mathcal{M}|$, the universal property of the free $\mathcal{T}$-model gives us a unique $\mathcal{T}$-homomorphism $f^{\ddagger} : \mathrm{Free}_{\mathcal{T}}(X) \to |\mathcal{M}|$ satisfying

$$f^{\ddagger}\,[\mathsf{return}\, x] = f\, x, \qquad\qquad f^{\ddagger}\,[\mathsf{op}(a, \kappa)] = \mathsf{op}_{\mathcal{M}}(a, f^{\ddagger} \circ \kappa).$$

A handler for a theory $\mathcal{T}$ in a language such as EFF amounts to a model $\mathcal{M}$ whose carrier $|\mathcal{M}|$ is the carrier $\mathrm{Free}_{\mathcal{T}'}(Y)$ of the free model for some other theory $\mathcal{T}'$, while the associated handling construct is the induced $\mathcal{T}$-homomorphism $\mathrm{Free}_{\mathcal{T}}(X) \to \mathrm{Free}_{\mathcal{T}'}(Y)$. Thus handling transforms computations with effects $\mathcal{T}$ to computations with effects $\mathcal{T}'$. There is however no restriction on how a handler implements an operation, in particular, it may use its continuation in an arbitrary fashion. We shall put the universal property of free models to good use as well, while making sure that the continuations are always used affinely.

#### 2.2 Runners

Much like monads, handlers are useful for simulating computational effects, because they allow us to transform $\mathcal{T}$-computations to $\mathcal{T}'$-computations. However, eventually there has to be a "top level" where such transformations cease and actual computational effects happen. For these we need another concept, known as *runners* [35]. Runners are equivalent to the concept of *comodels* [27,31], which are "just models in the opposite category", although one has to apply the motto correctly by using powers and co-powers where seemingly exponentials and products would do. Without getting into the intricacies, let us spell out the definition.

**Definition 1.** A *runner* $\mathcal{R}$ for a signature $\Sigma$ is given by a carrier set $|\mathcal{R}|$ together with, for each $\mathsf{op} \in \Sigma$, a *co-operation* $\overline{\mathsf{op}}_{\mathcal{R}} : A_{\mathsf{op}} \to (|\mathcal{R}| \Rightarrow B_{\mathsf{op}} \times |\mathcal{R}|)$.

Runners are usually defined to have co-operations in the equivalent uncurried form $\overline{\mathsf{op}}_{\mathcal{R}} : A_{\mathsf{op}} \times |\mathcal{R}| \to B_{\mathsf{op}} \times |\mathcal{R}|$, but that is less convenient for our purposes.

Runners may be defined more generally for theories $\mathcal{T}$, rather than just signatures, by requiring that the co-operations satisfy $\mathrm{Eq}_{\mathcal{T}}$. We shall have no use for these, although we expect no obstacles in incorporating them into our work.

A runner tells us what to do when an effectful computation reaches the top-level runtime environment. Think of $|\mathcal{R}|$ as the set of configurations of the runtime environment. Given the current configuration $c \in |\mathcal{R}|$, the operation $\mathsf{op}(a, \kappa)$ is executed as the corresponding co-operation $\overline{\mathsf{op}}_{\mathcal{R}}\, a\, c$ whose result

$(b, c') \in B_{\mathsf{op}} \times |\mathcal{R}|$ gives the result of the operation $b$ and the next runtime configuration $c'$. The continuation $\kappa\, b$ then proceeds in runtime configuration $c'$.

It is not too difficult to turn this idea into a mathematical model. For any $X$, the co-operations induce a $\Sigma$-structure $\mathcal{M}$ with $|\mathcal{M}| \stackrel{\text{def}}{=} \mathsf{St}_{|\mathcal{R}|}X = (|\mathcal{R}| \Rightarrow X \times |\mathcal{R}|)$ and operations $\mathsf{op}_{\mathcal{M}} : A_{\mathsf{op}} \times (B_{\mathsf{op}} \Rightarrow \mathsf{St}_{|\mathcal{R}|}X) \to \mathsf{St}_{|\mathcal{R}|}X$ given by

$$\mathsf{op}_{\mathcal{M}}(a, \kappa) \stackrel{\text{def}}{=} \lambda c \,.\, \kappa\,(\pi_1(\overline{\mathsf{op}}_{\mathcal{R}}\; a\, c))\,(\pi_2(\overline{\mathsf{op}}_{\mathcal{R}}\; a\, c)).$$

We may then use the universal property of the free $\Sigma$-model to obtain a $\Sigma$-homomorphism $\mathsf{r}_X : \mathrm{Tree}_\Sigma\,(X) \to \mathsf{St}_{|\mathcal{R}|}X$ satisfying the equations

$$\mathsf{r}_X(\mathsf{return}\, x) = \lambda c \,.\, (x, c), \qquad\qquad \mathsf{r}_X(\mathsf{op}(a, \kappa)) = \mathsf{op}_{\mathcal{M}}(a, \mathsf{r}_X \circ \kappa).$$

The map $\mathsf{r}_X$ precisely captures the idea that a runner *runs computations* by transforming (static) computation trees into state-passing maps. Note how in the above definition of $\mathsf{op}_{\mathcal{M}}$, the continuation $\kappa$ is used in a controlled way, as it appears precisely once as the head of the outermost application. In terms of programming, this corresponds to linear use in a tail-call position.

Runners are less ad-hoc than they may seem. First, notice that $\mathsf{op}_{\mathcal{M}}$ is just the composition of the co-operation $\overline{\mathsf{op}}_{\mathcal{R}}$ with the state monad's Kleisli extension of the continuation $\kappa$, and so is the standard way of turning *generic effects* into $\Sigma$-structures [26]. Second, the map $\mathsf{r}_X$ is the component at $X$ of a monad morphism $\mathsf{r} : \mathrm{Tree}_\Sigma\,(-) \to \mathsf{St}_{|\mathcal{R}|}$. Møgelberg & Staton [21], as well as Uustalu [35], showed that the passage from a runner $\mathcal{R}$ to the corresponding monad morphism $\mathsf{r}$ forms a one-to-one correspondence between the former and the latter.

As defined, runners are too restrictive a model of top-level computation, because the only effect available to co-operations is state, but in practice the runtime environment may also signal errors and perform other effects, by calling its own runtime environment. We are led to the following generalisation.

**Definition 2.** For a signature $\Sigma$ and monad $T$, a *$T$-runner* $\mathcal{R}$ for $\Sigma$, or just an *effectful runner*, is given by, for each $\mathsf{op} \in \Sigma$, a *co-operation* $\overline{\mathsf{op}}_{\mathcal{R}} : A_{\mathsf{op}} \to TB_{\mathsf{op}}$.

The correspondence between runners and monad morphisms still holds.

**Proposition 3.** *For a signature $\Sigma$ and a monad $T$, the monad morphisms $\mathrm{Tree}_\Sigma\,(-) \to T$ are in one-to-one correspondence with $T$-runners for $\Sigma$.*

*Proof.* This is an easy generalisation of the correspondence for ordinary runners. Let us fix a signature $\Sigma$, and a monad $T$ with unit $\eta$ and Kleisli extension $-^\dagger$.

Let $\mathcal{R}$ be a $T$-runner for $\Sigma$. For any set $X$, $\mathcal{R}$ induces a $\Sigma$-structure $\mathcal{M}$ with $|\mathcal{M}| \stackrel{\text{def}}{=} TX$ and $\mathsf{op}_{\mathcal{M}} : A_{\mathsf{op}} \times (B_{\mathsf{op}} \Rightarrow TX) \to TX$ defined as $\mathsf{op}_{\mathcal{M}}(a, \kappa) \stackrel{\text{def}}{=} \kappa^\dagger(\overline{\mathsf{op}}_R\; a)$. As before, the universal property of the free model $\mathrm{Tree}_\Sigma\,(X)$ provides a unique $\Sigma$-homomorphism $\mathsf{r}_X : \mathrm{Tree}_\Sigma\,(X) \to TX$, satisfying the equations

$$\mathsf{r}_X(\mathsf{return}\, x) = \eta_X(x), \qquad\qquad \mathsf{r}_X(\mathsf{op}(a, \kappa)) = \mathsf{op}_{\mathcal{M}}(a, \mathsf{r}_X \circ \kappa).$$

The maps $\mathsf{r}_X$ collectively give us the desired monad morphism $\mathsf{r}$ induced by $\mathcal{R}$.

Conversely, given a monad morphism $\theta : \mathrm{Tree}_\Sigma\,(-) \to T$, we may recover a $T$-runner $\mathcal{R}$ for $\Sigma$ by defining the co-operations as $\overline{\mathsf{op}}_{\mathcal{R}}\; a \stackrel{\text{def}}{=} \theta_{B_{\mathsf{op}}}(\mathsf{op}(a, \lambda b \,.\, \mathsf{return}\, b))$. It is not hard to check that we have described a one-to-one correspondence. □

## 3 Programming with runners

If ordinary runners are not general enough, the effectful ones are too general: parameterised by arbitrary monads $T$, they do not combine easily and they lack a clear notion of resource management. Thus, we now engineer more specific monads whose associated runners can be turned into a programming concept. While we give up complete generality, the monads presented below are still quite versatile, as they are parameterised by arbitrary algebraic signatures $\Sigma$, and so are extensible and support various combinations of effects.

### 3.1 The user and kernel monads

Effectful source code running inside a runtime environment is just one example of a more general phenomenon in which effectful computations are enveloped by a layer that provides a supervised access to external resources: a user process is controlled by a kernel, a web page by a browser, an operating system by hardware, or a virtual machine, etc. We shall adopt the parlance of software systems, and refer to the two layers generically as the *user* and *kernel* code. Since the two kinds of code need not, and will not, use the same effects, each will be described by its own algebraic theory and compute in its own monad.

We first address the kernel theory. Specifically, we look for an algebraic theory such that effectful runners for the induced monad satisfy the following desiderata:

1. Runners support management and controlled finalisation of resources.
2. Runners may use further external resources.
3. Runners may signal failure caused by unavoidable circumstances.

The totality of external resources available to user code appears as a stateful external environment, even though it has no direct access to it. Thus, kernel computations should carry state. We achieve this by incorporating into the kernel theory the operations $\mathsf{getenv}$ and $\mathsf{setenv}$, and equations for state from Example 1.

Apart from managing state, kernel code should have access to further effects, which may be true external effects, or some outer layer of runners. In either case, we should allow the kernel code to call operations from a given signature $\Sigma$.

Because kernel computations ought to be able to signal failure, we should include an exception mechanism. In practice, many programming languages and systems have two flavours of exceptions, variously called recoverable and fatal, checked and unchecked, exceptions and errors, etc. One kind, which we call just *exceptions*, is raised by kernel code when a situation requires special attention by user code. The other kind, which we call *signals*, indicates an unrecoverable condition that prevents normal execution of user code. These correspond precisely to the two standard ways of combining exceptions with state, namely the coproduct and the tensor of algebraic theories [11]. The coproduct simply adjoins exceptions $\mathsf{raise} : E \rightsquigarrow \mathbb{0}$ from Example 2 to the theory of state, while the tensor extends the theory of state with signals $\mathsf{kill} : S \rightsquigarrow \mathbb{0}$, together with equations

$$\mathsf{getenv}(\lambda c\,.\,\mathsf{kill}\,s) = \mathsf{kill}\,s, \qquad \mathsf{setenv}(c, \mathsf{kill}\,s) = \mathsf{kill}\,s. \tag{1}$$

These equations say that a signal discards state, which makes it unrecoverable.

To summarise, the *kernel theory* $\mathcal{K}_{\Sigma,E,S,C}$ contains operations from a signature $\Sigma$, as well as state operations $\mathsf{getenv} : \mathbb{1} \rightsquigarrow C$, $\mathsf{setenv} : C \rightsquigarrow \mathbb{1}$, exceptions $\mathsf{raise} : E \rightsquigarrow \mathbb{0}$, and signals $\mathsf{kill} : S \rightsquigarrow \mathbb{0}$, with equations for state from Example 1, equations (1) relating state and signals, and for each operation $\mathsf{op} \in \Sigma$, equations

$$\mathsf{getenv}(\lambda c\,.\,\mathsf{op}(a, \kappa\, c)) = \mathsf{op}(a, \lambda b\,.\,\mathsf{getenv}(\lambda c\,.\,\kappa\, c\, b)),$$
$$\mathsf{setenv}(c, \mathsf{op}(a, \kappa)) = \mathsf{op}(a, \lambda b\,.\,\mathsf{setenv}(c, \kappa\, b)),$$

expressing that external operations do not interact with kernel state. It is not difficult to see that $\mathcal{K}_{\Sigma,E,S,C}$ induces, up to isomorphism, the *kernel monad*

$$\mathsf{K}_{\Sigma,E,S,C} X \quad \stackrel{\text{def}}{=} \quad C \Rightarrow \mathrm{Tree}_{\Sigma}\,(((X + E) \times C) + S)\,.$$

How about user code? It can of course call operations from a signature $\Sigma$ (not necessarily the same as the kernel code), and because we intend it to handle exceptions, it might as well have the ability to raise them. However, user code knows nothing about signals and kernel state. Thus, we choose the *user theory* $\mathcal{U}_{\Sigma,E}$ to be the algebraic theory with operations $\Sigma$, exceptions $\mathsf{raise} : E \rightsquigarrow \mathbb{0}$, and no equations. This theory induces the *user monad* $\mathsf{U}_{\Sigma,E} X \stackrel{\text{def}}{=} \mathrm{Tree}_{\Sigma}\,(X + E)$.

#### 3.2 Runners as a programming construct

In this section, we turn the ideas presented so far into programming constructs. We strive for a realistic result, but when faced with several design options, we prefer simplicity and semantic clarity. We focus here on translating the central concepts, and postpone various details to §4, where we present a full calculus.

We codify the idea of user and kernel computations by having syntactic categories for each of them, as well as one for values. We use letters $M$, $N$ to indicate user computations, $K$, $L$ for kernel computations, and $V$, $W$ for values.

User and kernel code raise exceptions with operation raise, and catch them with exception handlers based on Benton and Kennedy's *exceptional syntax* [7],

$$\mathsf{try}\ M\ \mathsf{with}\ \{\mathsf{return}\, x \mapsto N, \ldots, \mathsf{raise}\, e \mapsto N_e, \ldots\},$$

and analogously for kernel code. The familiar binding construct $\mathsf{let}\ x = M\ \mathsf{in}\ N$ is simply shorthand for $\mathsf{try}\ M\ \mathsf{with}\ \{\mathsf{return}\, x \mapsto N, \ldots, \mathsf{raise}\, e \mapsto \mathsf{raise}\, e, \ldots\}$.

As a programming concept, a runner $R$ takes the form

$$\{(\mathsf{op}\, x \mapsto K_{\mathsf{op}})_{\mathsf{op} \in \Sigma}\}_C,$$

where each $K_{\mathsf{op}}$ is a kernel computation, with the variable $x$ bound in $K_{\mathsf{op}}$, so that each clause $\mathsf{op}\, x \mapsto K_{\mathsf{op}}$ determines a co-operation for the kernel monad. The subscript $C$ indicates the type of the state used by the kernel code $K_{\mathsf{op}}$.

The corresponding elimination form is a handling-like construct

$$\mathsf{using}\ R \,@\, V\ \mathsf{run}\ M\ \mathsf{finally}\ F, \tag{2}$$

which uses the co-operations of runner $R$ "at" initial kernel state $V$ to run user code $M$, and finalises its return value, exceptions, and signals with $F$, see (3) below. When user code $M$ calls an operation $\mathsf{op}$, the enveloping run construct runs the corresponding co-operation $K_{\mathsf{op}}$ of $R$. While doing so, $K_{\mathsf{op}}$ might raise exceptions. But not every exception makes sense for every operation, and so we assign to each operation $\mathsf{op}$ a set of exceptions $E_{\mathsf{op}}$ which the co-operations implementing it may raise, by augmenting its operation signature with $E_{\mathsf{op}}$, as

$$\mathsf{op} : A_{\mathsf{op}} \rightsquigarrow B_{\mathsf{op}} \,!\, E_{\mathsf{op}}.$$

An exception raised by the co-operation $K_{\mathsf{op}}$ propagates back to the operation call in the user code. Therefore, an operation call should have not only a continuation $x \,.\, M$ receiving a result, but also continuations $N_e$, one for each $e \in E_{\mathsf{op}}$,

$$\mathsf{op}(V, (x \,.\, M), (N_e)_{e \in E_{\mathsf{op}}}).$$

If $K_{\mathsf{op}}$ returns a value $b \in B_{\mathsf{op}}$, the execution proceeds as $M[b/x]$, and as $N_e$ if $K_{\mathsf{op}}$ raises an exception $e \in E_{\mathsf{op}}$. In examples, we use the generic versions of operations [26], written $\mathsf{op}\ V$, which pass on return values and re-raise exceptions.

One can pass exceptions back to operation calls also in a language with handlers, such as Eff, by changing the signatures of operations to $A_{\mathsf{op}} \rightsquigarrow B_{\mathsf{op}} + E_{\mathsf{op}}$, and implementing the exception mechanism by hand, so that every operation call is followed by a case distinction on $B_{\mathsf{op}} + E_{\mathsf{op}}$. One is reminded of how operating system calls communicate errors back to user code as exceptional values.

A co-operation $K_{\mathsf{op}}$ may also send a signal, in which case the rest of the user code $M$ is skipped and the control proceeds directly to the corresponding case of the finalisation part $F$ of the run construct (2), whose syntactic form is

$$\{\mathsf{return}\ x \mathbin{@} c \mapsto N, \ldots, \mathsf{raise}\ e \mathbin{@} c \mapsto N_e, \ldots, \mathsf{kill}\ s \mapsto N_s, \ldots\}. \tag{3}$$

Specifically, if $M$ returns a value $v$, then $N$ is evaluated with $x$ bound to $v$ and $c$ to the final kernel state; if $M$ raises an exception $e$ (either directly or indirectly via a co-operation of $R$), then $N_e$ is executed, again with $c$ bound to the final kernel state; and if a co-operation of $R$ sends a signal $s$, then $N_s$ is executed.

*Example 4.* In anticipation of setting up the complete calculus we show how one can work with files. The language implementors can provide an operation open which opens a file for writing and returns its file handle, an operation close which closes a file handle, and a runner fileIO that implements writing. Let us further suppose that fileIO may raise an exception QuotaExceeded if a write exceeds the user disk quota, and send a signal IOError if an unrecoverable external error occurs. The following code illustrates how to guarantee proper closing of the file:

```
using fileIO @ (open "hello.txt") run
  write "Hello, world."
finally {
  return x @ fh → close fh,
  raise QuotaExceeded @ fh → close fh,
  kill IOError → return () }
```

Notice that the user code does not have direct access to the file handle. Instead, the runner holds it in its state, where it is available to the co-operation that implements write. The finalisation block gets access to the file handle upon successful completion and raised exception, so it can close the file, but when a signal happens the finalisation cannot close the file, nor should it attempt to do so.

We also mention that the code "cheats" by placing the call to open in a position where a value is expected. We should have let-bound the file handle returned by open outside the run construct, which would make it clear that opening the file happens *before* this construct (and that open is *not* handled by the finalisation), but would also expose the file handle. Since there are clear advantages to keeping the file handle inaccessible, a realistic language should accept the above code and hoist computations from value positions automatically.

### 4 A calculus for programming with runners

Inspired by the semantic notion of runners and the ideas of the previous section, we now present a calculus for programming with co-operations and runners, called $\lambda_{\mathsf{coop}}$. It is a low-level fine-grain call-by-value calculus [19], and as such could inspire an intermediate language that a high-level language is compiled to.

#### 4.1 Types

The types of $\lambda_{\mathsf{coop}}$ are shown in Fig. 1. The *ground types* contain *base types*, and are closed under finite sums and products. These are used in operation signatures and as types of kernel state. (Allowing arbitrary types in either of these entails substantial complications that can be dealt with but are tangential to our goals.) Ground types can also come with corresponding constant symbols $\mathsf{f}$, each associated with a fixed *constant signature* $\mathsf{f} : (A_1, \ldots, A_n) \to B$.

We assume a supply of operation symbols $\mathcal{O}$, exception names $\mathcal{E}$, and signal names $\mathcal{S}$. Each operation symbol $\mathsf{op} \in \mathcal{O}$ is equipped with an *operation signature* $A_{\mathsf{op}} \leadsto B_{\mathsf{op}} \,!\, E_{\mathsf{op}}$, which specifies its parameter type $A_{\mathsf{op}}$ and arity type $B_{\mathsf{op}}$, and the exceptions $E_{\mathsf{op}}$ that the corresponding co-operations may raise in runners.

The *value types* extend ground types with two function types, and a type of runners. The *user function type* $X \to Y \,!\, (\Sigma, E)$ classifies functions taking arguments of type $X$ to computations classified by the *user (computation) type* $Y \,!\, (\Sigma, E)$, i.e., those that return values of type $Y$, and may call operations $\Sigma$ and raise exceptions $E$. Similarly, the *kernel function type* $X \to Y \,\lightning\, (\Sigma, E, S, C)$ classifies functions taking arguments of type $X$ to computations classified by the *kernel (computation) type* $Y \,\lightning\, (\Sigma, E, S, C)$, i.e., those that return values of type $Y$, and may call operations $\Sigma$, raise exceptions $E$, send signals $S$, and use state of type $C$. We note that the ingredients for user and kernel types correspond precisely to the parameters of the user monad $\mathsf{U}_{\Sigma,E}$ and the kernel monad $\mathsf{K}_{\Sigma,E,S,C}$ from §3.1. Finally, the *runner type* $\Sigma \Rightarrow (\Sigma', S, C)$ classifies runners that implement co-operations for the operations $\Sigma$ as kernel computations which use operations $\Sigma'$, send signals $S$, and use state of type $C$.

$$
\begin{array}{rcll}
\text{Ground type } A, B, C & ::= & \mathsf{b} & \text{base type} \\
& | & \mathsf{unit} & \text{unit type} \\
& | & \mathsf{empty} & \text{empty type} \\
& | & A \times B & \text{product type} \\
& | & A + B & \text{sum type} \\
\text{Constant signature:} & & \mathsf{f} : (A_1, \ldots, A_n) \to B & \\
\text{Signature } \Sigma & ::= & \{\mathsf{op}_1, \mathsf{op}_2, \ldots, \mathsf{op}_n\} \subset \mathcal{O} & \\
\text{Exception set } E & ::= & \{e_1, e_2, \ldots, e_n\} \subset \mathcal{E} & \\
\text{Signal set } S & ::= & \{s_1, s_2, \ldots, s_n\} \subset \mathcal{S} & \\
\text{Operation signature:} & & \mathsf{op} : A_{\mathsf{op}} \rightsquigarrow B_{\mathsf{op}} \mathbin{!} E_{\mathsf{op}} & \\
\text{Value type } X, Y, Z & ::= & A & \text{ground type} \\
& | & X \times Y & \text{product type} \\
& | & X + Y & \text{sum type} \\
& | & X \to Y \mathbin{!} \mathcal{U} & \text{user function type} \\
& | & X \to Y \mathbin{\lightning} \mathcal{K} & \text{kernel function type} \\
& | & \Sigma \Rightarrow (\Sigma', S, C) & \text{runner type} \\
\text{User (computation) type:} & & X \mathbin{!} \mathcal{U} \quad \text{where } \mathcal{U} = (\Sigma, E) & \\
\text{Kernel (computation) type:} & & X \mathbin{\lightning} \mathcal{K} \quad \text{where } \mathcal{K} = (\Sigma, E, S, C) &
\end{array}
$$

**Fig. 1.** The types of $\lambda_{\mathsf{coop}}$.

#### 4.2 Values and computations

The syntax of terms is shown in Fig. 2. The usual fine-grain call-by-value stratification of terms into pure values and effectful computations is present, except that we further distinguish between *user* and *kernel* computations.

**Values** Among the values are variables, constants for ground types, and constructors for sums and products. There are two kinds of functions, for abstracting over user and kernel computations. A *runner* is a value of the form

$$\{(\mathsf{op}\, x \mapsto K_{\mathsf{op}})_{\mathsf{op} \in \Sigma}\}_C.$$

It implements co-operations for operations $\mathsf{op}$ as kernel computations $K_{\mathsf{op}}$, with $x$ bound in $K_{\mathsf{op}}$. The type annotation $C$ specifies the type of the state that $K_{\mathsf{op}}$ uses. Note that $C$ ranges over ground types, a restriction that allows us to define a naive set-theoretic semantics. We sometimes omit these type annotations.

**User and kernel computations** The user and kernel computations both have pure computations, function application, exception raising and handling, stan-

**Values**

$$
\begin{array}{lll}
V, W & ::= x & \text{variable} \\
& \mid\ \mathsf{f}(V_1, \ldots, V_n) & \text{ground constant} \\
& \mid\ () & \text{unit} \\
& \mid\ (V, W) & \text{pair} \\
& \mid\ \mathsf{inl}_{X,Y}\, V \ \mid\ \mathsf{inr}_{X,Y}\, V & \text{injection} \\
& \mid\ \mathsf{fun}\ (x : X) \mapsto M & \text{user function} \\
& \mid\ \mathsf{funK}\ (x : X) \mapsto K & \text{kernel function} \\
& \mid\ \{(\mathsf{op}\, x \mapsto K_{\mathsf{op}})_{\mathsf{op} \in \Sigma}\}_C & \text{runner}
\end{array}
$$

**User computations**

$$
\begin{array}{lll}
M, N & ::= \mathsf{return}\ V & \text{value} \\
& \mid\ V\, W & \text{application} \\
& \mid\ \mathsf{try}\ M\ \mathsf{with}\ \{\mathsf{return}\, x \mapsto N, (\mathsf{raise}\, e \mapsto N_e)_{e \in E}\} & \text{exception handler} \\
& \mid\ \mathsf{match}\ V\ \mathsf{with}\ \{(x, y) \mapsto M\} & \text{product elimination} \\
& \mid\ \mathsf{match}\ V\ \mathsf{with}\ \{\}_X & \text{empty elimination} \\
& \mid\ \mathsf{match}\ V\ \mathsf{with}\ \{\mathsf{inl}\, x \mapsto M, \mathsf{inr}\, y \mapsto N\} & \text{sum elimination} \\
& \mid\ \mathsf{op}_X(V, (x \,.\, M), (N_e)_{e \in E_{\mathsf{op}}}) & \text{operation call} \\
& \mid\ \mathsf{raise}_X\, e & \text{raise exception} \\
& \mid\ \mathsf{using}\ V \mathbin{@} W\ \mathsf{run}\ M\ \mathsf{finally}\ F & \text{running user code} \\
& \mid\ \mathsf{kernel}\ K \mathbin{@} W\ \mathsf{finally}\ F & \text{switch to kernel mode}
\end{array}
$$

$$
F ::= \{\mathsf{return}\, x \mathbin{@} c \mapsto N, (\mathsf{raise}\, e \mathbin{@} c \mapsto N_e)_{e \in E}, (\mathsf{kill}\, s \mapsto N_s)_{s \in S}\}
$$

**Kernel computations**

$$
\begin{array}{lll}
K, L & ::= \mathsf{return}_C\ V & \text{value} \\
& \mid\ V\, W & \text{application} \\
& \mid\ \mathsf{try}\ K\ \mathsf{with}\ \{\mathsf{return}\, x \mapsto L, (\mathsf{raise}\, e \mapsto L_e)_{e \in E}\} & \text{exception handler} \\
& \mid\ \mathsf{match}\ V\ \mathsf{with}\ \{(x, y) \mapsto K\} & \text{product elimination} \\
& \mid\ \mathsf{match}\ V\ \mathsf{with}\ \{\}_{X @ C} & \text{empty elimination} \\
& \mid\ \mathsf{match}\ V\ \mathsf{with}\ \{\mathsf{inl}\, x \mapsto K, \mathsf{inr}\, y \mapsto L\} & \text{sum elimination} \\
& \mid\ \mathsf{op}_X(V, (x \,.\, K), (L_e)_{e \in E_{\mathsf{op}}}) & \text{operation call} \\
& \mid\ \mathsf{raise}_{X @ C}\, e & \text{raise exception} \\
& \mid\ \mathsf{kill}_{X @ C}\, s & \text{send signal} \\
& \mid\ \mathsf{getenv}_C(c \,.\, K) & \text{get kernel state} \\
& \mid\ \mathsf{setenv}(V, K) & \text{set kernel state} \\
& \mid\ \mathsf{user}\ M\ \mathsf{with}\ \{\mathsf{return}\, x \mapsto K, (\mathsf{raise}\, e \mapsto L_e)_{e \in E}\} & \text{switch to user mode}
\end{array}
$$

**Fig. 2.** Values, user computations, and kernel computations of $\lambda_{\mathsf{coop}}$.

dard elimination forms, and operation calls. Note that the typing annotations on some of these differ according to their mode. For instance, a user operation call is annotated with the result type $X$, whereas the annotation $X \mathbin{@} C$ on a kernel operation call also specifies the kernel state type $C$.

The binding construct $\mathsf{let}_{X!E}\ x = M\ \mathsf{in}\ N$ is not part of the syntax, but is an abbreviation for $\mathsf{try}\ M\ \mathsf{with}\ \{\mathsf{return}\ x \mapsto N, (\mathsf{raise}\ e \mapsto \mathsf{raise}_X\ e)_{e \in E}\}$, and there is an analogous one for kernel computations. We often drop the annotation $X!E$.

Some computations are specific to one or the other mode. Only the kernel mode may send a signal with $\mathsf{kill}$, and manipulate state with $\mathsf{getenv}$ and $\mathsf{setenv}$, but only the user mode has the $\mathsf{run}$ construct from §3.2. Finally, each mode has the ability to "context switch" to the other one. The kernel computation

$$\mathsf{user}\ M\ \mathsf{with}\ \{\mathsf{return}\ x \mapsto K, (\mathsf{raise}\ e \mapsto L_e)_{e \in E}\}$$

runs a user computation $M$ and handles the returned value and leftover exceptions with kernel computations $K$ and $L_e$. Conversely, the user computation

$$\mathsf{kernel}\ K \mathbin{@} W\ \mathsf{finally}\ \{x \mathbin{@} c \mapsto M, (\mathsf{raise}\ e \mathbin{@} c \mapsto N_e)_{e \in E}, (\mathsf{kill}\ s \mapsto N_s)_{s \in S}\}$$

runs kernel computation $K$ with initial state $W$, and handles the returned value, and leftover exceptions and signals with user computations $M$, $N_e$, and $N_s$.

#### 4.3 Type system

We equip $\lambda_{\mathsf{coop}}$ with a type system akin to type and effect systems for algebraic effects and handlers [3,7,12]. We are experimenting with resource control, so it makes sense for the type system to tightly control resources. Consequently, our effect system does not allow effects to be implicitly propagated outwards.

In §4.1, we assumed that each operation $\mathsf{op} \in \mathcal{O}$ is equipped with some fixed operation signature $\mathsf{op} : A_{\mathsf{op}} \leadsto B_{\mathsf{op}} \mathbin{!} E_{\mathsf{op}}$. We also assumed a fixed constant signature $\mathsf{f} : (A_1, \ldots, A_n) \to B$ for each ground constant $\mathsf{f}$. We consider this information to be part of the type system and say no more about it.

Values, user computations, and kernel computations each have a corresponding *typing judgement* form and a *subtyping relation*, given by

$$\begin{array}{lll} \Gamma \vdash V : X, & \Gamma \vdash M : X \mathbin{!} \mathcal{U}, & \Gamma \vdash K : X \mathbin{\lightning} \mathcal{K}, \\ X \sqsubseteq Y, & X \mathbin{!} \mathcal{U} \sqsubseteq Y \mathbin{!} \mathcal{V}, & X \mathbin{\lightning} \mathcal{K} \sqsubseteq Y \mathbin{\lightning} \mathcal{L}, \end{array}$$

where $\Gamma$ is a *typing context* $x_1 : X_1, \ldots, x_n : X_n$. The effect information is an over-approximation, i.e., $M$ and $K$ employ *at most* the effects described by $\mathcal{U}$ and $\mathcal{K}$. The complete rules for these judgements are given in the online appendix. We comment here only on the rules that are peculiar to $\lambda_{\mathsf{coop}}$, see Fig. 3.

Subtyping of ground types Sub-Ground is trivial, as it relates only equal types. Subtyping of runners Sub-Runner and kernel computations Sub-Kernel requires equality of the kernel state types $C$ and $C'$ because state is used invariantly in the kernel monad. We leave it for future work to replace $C \equiv C'$ with a *lens* [10] from $C'$ to $C$, i.e., maps $C' \to C$ and $C' \times C \to C'$ satisfying state

$$\textsc{Sub-Ground}\quad \frac{}{A \sqsubseteq A}$$

$$\textsc{Sub-Runner}\quad \frac{\Sigma'_1 \subseteq \Sigma_1 \qquad \Sigma_2 \subseteq \Sigma'_2 \qquad S \subseteq S' \qquad C \equiv C'}{\Sigma_1 \Rightarrow (\Sigma_2, S, C) \sqsubseteq \Sigma'_1 \Rightarrow (\Sigma'_2, S', C')}$$

$$\textsc{Sub-Kernel}\quad \frac{X \sqsubseteq X' \qquad \Sigma \subseteq \Sigma' \qquad E \subseteq E' \qquad S \subseteq S' \qquad C \equiv C'}{X \mathbin{\text{↯}} (\Sigma, E, S, C) \sqsubseteq X' \mathbin{\text{↯}} (\Sigma', E', S', C')}$$

$$\textsc{TyUser-Try}\quad \frac{\Gamma \vdash M : X \,!\, (\Sigma, E) \qquad \Gamma, x \,{:}\, X \vdash N : Y \,!\, (\Sigma, E') \qquad \big(\Gamma \vdash N_e : Y \,!\, (\Sigma, E')\big)_{e \in E}}{\Gamma \vdash \mathsf{try}\ M\ \mathsf{with}\ \{\mathsf{return}\ x \mapsto N, (\mathsf{raise}\ e \mapsto N_e)_{e \in E}\} : Y \,!\, (\Sigma, E')}$$

$$\textsc{TyUser-Run}\quad \frac{\begin{array}{c} F \equiv \{\mathsf{return}\ x \mathbin{@} c \mapsto N, (\mathsf{raise}\ e \mathbin{@} c \mapsto N_e)_{e \in E}, (\mathsf{kill}\ s \mapsto N_s)_{s \in S}\} \\ \Gamma \vdash V : \Sigma \Rightarrow (\Sigma', S, C) \qquad \Gamma \vdash W : C \\ \Gamma \vdash M : X \,!\, (\Sigma, E) \qquad \Gamma, x \,{:}\, X, c \,{:}\, C \vdash N : Y \,!\, (\Sigma', E') \\ \big(\Gamma, c \,{:}\, C \vdash N_e : Y \,!\, (\Sigma', E')\big)_{e \in E} \qquad \big(\Gamma \vdash N_s : Y \,!\, (\Sigma', E')\big)_{s \in S} \end{array}}{\Gamma \vdash \mathsf{using}\ V \mathbin{@} W\ \mathsf{run}\ M\ \mathsf{finally}\ F : Y \,!\, (\Sigma', E')}$$

$$\textsc{TyUser-Op}\quad \frac{\begin{array}{c} \mathcal{U} \equiv (\Sigma, E) \qquad \mathsf{op} \in \Sigma \qquad \Gamma \vdash V : A_{\mathsf{op}} \\ \Gamma, x \,{:}\, B_{\mathsf{op}} \vdash M : X \,!\, \mathcal{U} \qquad \big(\Gamma \vdash N_e : X \,!\, \mathcal{U}\big)_{e \in E_{\mathsf{op}}} \end{array}}{\Gamma \vdash \mathsf{op}_X(V, (x \,.\, M), (N_e)_{e \in E_{\mathsf{op}}}) : X \,!\, \mathcal{U}}$$

$$\textsc{TyKernel-Op}\quad \frac{\begin{array}{c} \mathcal{K} \equiv (\Sigma, E, S, C) \qquad \mathsf{op} \in \Sigma \qquad \Gamma \vdash V : A_{\mathsf{op}} \\ \Gamma, x \,{:}\, B_{\mathsf{op}} \vdash K : X \mathbin{\text{↯}} \mathcal{K} \qquad \big(\Gamma \vdash L_e : X \mathbin{\text{↯}} \mathcal{K}\big)_{e \in E_{\mathsf{op}}} \end{array}}{\Gamma \vdash \mathsf{op}_X(V, (x \,.\, K), (L_e)_{e \in E_{\mathsf{op}}}) : X \mathbin{\text{↯}} \mathcal{K}}$$

$$\textsc{TyUser-Kernel}\quad \frac{\begin{array}{c} F \equiv \{\mathsf{return}\ x \mathbin{@} c \mapsto N, (\mathsf{raise}\ e \mathbin{@} c \mapsto N_e)_{e \in E}, (\mathsf{kill}\ s \mapsto N_s)_{s \in S}\} \\ \Gamma \vdash K : X \mathbin{\text{↯}} (\Sigma, E, S, C) \qquad \Gamma \vdash W : C \qquad \Gamma, x \,{:}\, X, c \,{:}\, C \vdash N : Y \,!\, (\Sigma, E') \\ \big(\Gamma, c \,{:}\, C \vdash N_e : Y \,!\, (\Sigma, E')\big)_{e \in E} \qquad \big(\Gamma \vdash N_s : Y \,!\, (\Sigma, E')\big)_{s \in S} \end{array}}{\Gamma \vdash \mathsf{kernel}\ K \mathbin{@} W\ \mathsf{finally}\ F : Y \,!\, (\Sigma, E')}$$

$$\textsc{TyKernel-User}\quad \frac{\begin{array}{c} \mathcal{K} \equiv (\Sigma, E', S, C) \qquad \Gamma \vdash M : X \,!\, (\Sigma, E) \\ \Gamma, x \,{:}\, X \vdash K : Y \mathbin{\text{↯}} \mathcal{K} \qquad \big(\Gamma \vdash L_e : Y \mathbin{\text{↯}} \mathcal{K}\big)_{e \in E} \end{array}}{\Gamma \vdash \mathsf{user}\ M\ \mathsf{with}\ \{\mathsf{return}\ x \mapsto K, (\mathsf{raise}\ e \mapsto L_e)_{e \in E}\} : Y \mathbin{\text{↯}} \mathcal{K}}$$

**Fig. 3.** Selected typing and subtyping rules.

equations analogous to Example 1. It has been observed [24,31] that such a lens in fact amounts to an ordinary runner for $C$-valued state.

The rules TyUser-Op and TyKernel-Op govern operation calls, where we have a success continuation which receives a value returned by a co-operation, and exceptional continuations which receive exceptions raised by co-operations.

The rule TyUser-Run requires that the runner $V$ implements *all* the operations $M$ can use, meaning that operations are *not* implicitly propagated outside a run block (which is different from how handlers are sometimes implemented). Of course, the co-operations of the runner may call further external operations, as recorded by the signature $\Sigma'$. Similarly, we require the finally block $F$ to intercept all exceptions and signals that might be produced by the co-operations of $V$ or the user code $M$. Such strict control is exercised throughout. For example, in TyUser-Run, TyUser-Kernel, and TyKernel-User we catch all the exceptions and signals that the code might produce. One should judiciously relax these requirements in a language that is presented to the programmer, and allow re-raising and re-sending clauses to be automatically inserted.

#### 4.4 Equational theory

We present $\lambda_{\mathsf{coop}}$ as an *equational calculus*, i.e., the interactions between its components are described by equations. Such a presentation makes it easy to reason about program equivalence. There are three equality judgements

$$\Gamma \vdash V \equiv W : X, \qquad \Gamma \vdash M \equiv N : X \,!\, \mathcal{U}, \qquad \Gamma \vdash K \equiv L : X \,!\, \mathcal{K}.$$

It is presupposed that we only compare well-typed expressions with the indicated types. For the most part, the context and the type annotation on judgements will play no significant role, and so we shall drop them whenever possible.

We comment on the computational equations for constructs characteristic of $\lambda_{\mathsf{coop}}$, and refer the reader to the online appendix for other equations. When read left-to-right, these equations explain the operational meaning of programs.

Of the three equations for run, the first two specify that returned values and raised exceptions are handled by the corresponding clauses,

$$\begin{aligned} \mathsf{using}\ V \mathbin{@} W\ \mathsf{run}\ (\mathsf{return}\ V')\ \mathsf{finally}\ F &\equiv N[V'/x, W/c], \\ \mathsf{using}\ V \mathbin{@} W\ \mathsf{run}\ (\mathsf{raise}_X\ e)\ \mathsf{finally}\ F &\equiv N_e[W/c], \end{aligned}$$

where $F \stackrel{\text{def}}{=} \{\mathsf{return}\ x \mathbin{@} c \mapsto N, (\mathsf{raise}\ e \mathbin{@} c \mapsto N_e)_{e \in E}, (\mathsf{kill}\ s \mapsto N_s)_{s \in S}\}$. The third equation below relates running an operation $\mathsf{op}$ with executing the corresponding co-operation $K_{\mathsf{op}}$, where $R$ stands for the runner $\{(\mathsf{op}\ x \mapsto K_{\mathsf{op}})_{\mathsf{op} \in \Sigma}\}_C$:

$$\begin{aligned} &\mathsf{using}\ R \mathbin{@} W\ \mathsf{run}\ (\mathsf{op}_X(V, (x\,.\,M), (N'_{e'})_{e' \in E_{\mathsf{op}}}))\ \mathsf{finally}\ F \equiv \\ &\qquad \mathsf{kernel}\ K_{\mathsf{op}}[V/x] \mathbin{@} W\ \mathsf{finally} \\ &\qquad\qquad \big\{\mathsf{return}\ x \mathbin{@} c' \mapsto (\mathsf{using}\ R \mathbin{@} c'\ \mathsf{run}\ M\ \mathsf{finally}\ F), \\ &\qquad\qquad\ \big(\mathsf{raise}\ e' \mathbin{@} c' \mapsto (\mathsf{using}\ R \mathbin{@} c'\ \mathsf{run}\ N'_{e'}\ \mathsf{finally}\ F)\big)_{e' \in E_{\mathsf{op}}}, \\ &\qquad\qquad\ (\mathsf{kill}\ s \mapsto N_s)_{s \in S}\ \big\} \end{aligned}$$

Because $K_{\mathsf{op}}$ is kernel code, it is executed in kernel mode, whose finally clauses specify what happens afterwards: if $K_{\mathsf{op}}$ returns a value, or raises an exception, execution continues with a suitable continuation, with $R$ wrapped around it; and if $K_{\mathsf{op}}$ sends a signal, the corresponding finalisation code from $F$ is evaluated.

The next bundle describes how kernel code is executed within user code:

$$\begin{aligned}
\mathsf{kernel}\ (\mathsf{return}_C\, V) \mathbin{@} W\ \mathsf{finally}\ F &\equiv N[V/x, W/c],\\
\mathsf{kernel}\ (\mathsf{raise}_{X@C}\, e) \mathbin{@} W\ \mathsf{finally}\ F &\equiv N_e[W/c],\\
\mathsf{kernel}\ (\mathsf{kill}_{X@C}\, s) \mathbin{@} W\ \mathsf{finally}\ F &\equiv N_s,\\
\mathsf{kernel}\ (\mathsf{getenv}_C(c\,.\,K)) \mathbin{@} W\ \mathsf{finally}\ F &\equiv \mathsf{kernel}\ K[W/c] \mathbin{@} W\ \mathsf{finally}\ F,\\
\mathsf{kernel}\ (\mathsf{setenv}(V, K)) \mathbin{@} W\ \mathsf{finally}\ F &\equiv \mathsf{kernel}\ K \mathbin{@} V\ \mathsf{finally}\ F.
\end{aligned}$$

We also have an equation stating that an operation called in kernel mode propagates out to user mode, with its continuations wrapped in kernel mode:

$$\begin{aligned}
&\mathsf{kernel}\ \mathsf{op}_X(V, (x\,.\,K), (L_{e'})_{e' \in E}) \mathbin{@} W\ \mathsf{finally}\ F \equiv\\
&\qquad \mathsf{op}_X(V, (x\,.\,\mathsf{kernel}\ K \mathbin{@} W\ \mathsf{finally}\ F), (\mathsf{kernel}\ L_{e'} \mathbin{@} W\ \mathsf{finally}\ F)_{e' \in E}).
\end{aligned}$$

Similar equations govern execution of user computations in kernel mode.

The remaining equations include standard $\beta\eta$-equations for exception handling [7], deconstruction of products and sums, algebraicity equations for operations [33], and the equations of kernel theory from §3.1, describing how getenv and setenv work, and how they interact with signals and other operations.

### 5 Denotational semantics

We provide a coherent denotational semantics for $\lambda_{\mathsf{coop}}$, and prove it sound with respect to the equational theory given in §4.4. Having eschewed all forms of recursion, we may afford to work simply over the category of sets and functions, while noting that there is no obstacle to incorporating recursion at all levels and switching to domain theory, similarly to the treatment of effect handlers in [3].

#### 5.1 Semantics of types

The meaning of terms is most naturally defined by structural induction on their typing derivations, which however are not unique in $\lambda_{\mathsf{coop}}$ due to subsumption rules. Thus we must worry about devising a *coherent* semantics, i.e., one in which all derivations of a judgement get the same meaning. We follow prior work on the semantics of effect systems for handlers [3], and proceed by first giving a *skeletal* semantics of $\lambda_{\mathsf{coop}}$ in which derivations are manifestly unique because the effect information is unrefined. We then use the skeletal semantics as the frame upon which rests a refinement-style coherent semantics of the effectful types of $\lambda_{\mathsf{coop}}$.

The *skeletal* types are like $\lambda_{\mathsf{coop}}$'s types, but with all effect information erased. In particular, the ground types $A$, and hence the kernel state types $C$, do not change as they contain no effect information. The skeletal value types are

$$P, Q \ ::= \ A \mid \mathsf{unit} \mid \mathsf{empty} \mid P \times Q \mid P + Q \mid P \to Q! \mid P \to Q \mathbin{\lightning} C \mid \mathsf{runner}\, C.$$

The skeletal versions of the user and kernel types are $P!$ and $P \lightning C$, respectively. It is best to think of the skeletal types as ML-style types which implicitly over-approximate effect information by "any effect is possible", an idea which is mathematically expressed by their semantics, as explained below.

First of all, the semantics of ground types is straightforward. One only needs to provide sets denoting the base types $\mathsf{b}$, after which the ground types receive the standard set-theoretic meaning, as given in Fig. 4.

Recall that $\mathcal{O}$, $\mathcal{S}$, and $\mathcal{E}$ are the sets of all operations, signals, and exceptions, and that each $\mathsf{op} \in \mathcal{O}$ has a signature $\mathsf{op} : A_{\mathsf{op}} \rightsquigarrow B_{\mathsf{op}} \,!\, E_{\mathsf{op}}$. Let us additionally assume that there is a distinguished operation $\text{⏊} \in \mathcal{O}$ with signature $\text{⏊} : \mathbb{1} \rightsquigarrow \mathbb{0} \,!\, \mathbb{0}$ (otherwise we adjoin it to $\mathcal{O}$). It ensures that the denotations of skeletal user and kernel types are *pointed* sets, while operationally ⏊ indicates a *runtime error*.

Next, we define the *skeletal user and kernel monads* as

$$\mathsf{U}^{\mathsf{s}} X \stackrel{\text{def}}{=} \mathsf{U}_{\mathcal{O},\mathcal{E}} X = \mathrm{Tree}_{\mathcal{O}}\,(X + \mathcal{E})\,,$$

$$\mathsf{K}^{\mathsf{s}}_{C} X \stackrel{\text{def}}{=} \mathsf{K}_{\mathcal{O},\mathcal{E},\mathcal{S},C} X = (C \Rightarrow \mathrm{Tree}_{\mathcal{O}}\,((X + \mathcal{E}) \times C + \mathcal{S})),$$

and $\mathsf{Runner}^{\mathsf{s}}\, C$ as the set of all *skeletal runners* $\mathcal{R}$ *(with state* $C$*)*, which are families of co-operations $\{\overline{\mathsf{op}}_{\mathcal{R}} : [\![A_{\mathsf{op}}]\!] \to \mathsf{K}_{\mathcal{O},E_{\mathsf{op}},\mathcal{S},C} [\![B_{\mathsf{op}}]\!]\}_{\mathsf{op} \in \mathcal{O}}$. Note that $\mathsf{K}_{\mathcal{O},E_{\mathsf{op}},\mathcal{S},C}$ is a coproduct [11] of monads $C \Rightarrow \mathrm{Tree}_{\mathcal{O}}\,(- \times C + \mathcal{S})$ and $\mathsf{Exc}_{E_{\mathsf{op}}}$, and thus the skeletal runners are the effectful runners for the former monad, so long as we read the effectful signatures $\mathsf{op} : A_{\mathsf{op}} \rightsquigarrow B_{\mathsf{op}} \,!\, E_{\mathsf{op}}$ as ordinary algebraic ones $\mathsf{op} : A_{\mathsf{op}} \rightsquigarrow B_{\mathsf{op}} + E_{\mathsf{op}}$. While there is no semantic difference between the two readings, there is one of intention: $\mathsf{K}_{\mathcal{O},E_{\mathsf{op}},\mathcal{S},C} [\![B_{\mathsf{op}}]\!]$ is a kernel computation that (apart from using state and sending signals) returns values of type $B_{\mathsf{op}}$ and raises exceptions $E_{\mathsf{op}}$, whereas $C \Rightarrow \mathrm{Tree}_{\mathcal{O}}\,(([\![B_{\mathsf{op}}]\!] + E_{\mathsf{op}}) \times C + \mathcal{S})$ returns values of type $B_{\mathsf{op}} + E_{\mathsf{op}}$ and raises no exceptions. We prefer the former, as it reflects our treatment of exceptions as a control mechanism rather than exceptional values.

These ingredients suffice for the denotation of skeletal types as sets, as given in Fig. 4. The user and kernel skeletal types are interpreted using the respective skeletal monads, and hence the two function types as Kleisli exponentials.

We proceed with the semantics of effectful types. The *skeleton* of a value type $X$ is the skeletal type $X^{\mathsf{s}}$ obtained by removing all effect information, and similarly for user and kernel types, see Fig. 5. We interpret a value type $X$ as a subset $[\![[X]\!]] \subseteq [\![X^{\mathsf{s}}]\!]$ of the denotation of its skeleton, and similarly for user and computation types. In other words, we treat the effectful types as *refinements* of their skeletons. For this, we define the operation $(X_0, X_1) \Rrightarrow (Y_0, Y_1)$, for any $X_0 \subseteq X_1$ and $Y_0 \subseteq Y_1$, as the set of maps $X_1 \to Y_1$ restricted to $X_0 \to Y_0$:

$$(X_0, X_1) \Rrightarrow (Y_0, Y_1) \stackrel{\text{def}}{=} \{f : X_1 \to Y_1 \mid \forall x \in X_0 \,.\, f(x) \in Y_0\}.$$

Next, observe that the user and the kernel monads preserve subset inclusions, in the sense that $\mathsf{U}_{\Sigma,E} X \subseteq \mathsf{U}_{\Sigma',E'} X'$ and $\mathsf{K}_{\Sigma,E,S,C} X \subseteq \mathsf{K}_{\Sigma',E',S',C} X'$ if $\Sigma \subseteq \Sigma'$, $E \subseteq E'$, $S \subseteq S'$, and $X \subseteq X'$. In particular, we always have $\mathsf{U}_{\Sigma,E} X \subseteq \mathsf{U}^{\mathsf{s}} X$ and $\mathsf{K}_{\Sigma,E,S,C} X \subseteq \mathsf{K}^{\mathsf{s}}_{C} X$. Finally, let $\mathsf{Runner}_{\Sigma,\Sigma',S}\, C \subseteq \mathsf{Runner}^{\mathsf{s}}\, C$ be the subset of those runners $\mathcal{R}$ whose co-operations for $\Sigma$ factor through $\mathsf{K}_{\Sigma',E_{\mathsf{op}},S,C}$, i.e., $\overline{\mathsf{op}}_{\mathcal{R}} : [\![A_{\mathsf{op}}]\!] \to \mathsf{K}_{\Sigma',E_{\mathsf{op}},S,C} [\![B_{\mathsf{op}}]\!] \subseteq \mathsf{K}_{\mathcal{O},E_{\mathsf{op}},\mathcal{S},C} [\![B_{\mathsf{op}}]\!]$, for each $\mathsf{op} \in \Sigma$.

**Ground types**

$$[\![\mathsf{b}]\!] \stackrel{\text{def}}{=} \cdots \qquad [\![\mathsf{unit}]\!] \stackrel{\text{def}}{=} \mathbb{1} \qquad [\![\mathsf{empty}]\!] \stackrel{\text{def}}{=} \mathbb{0}$$

$$[\![A \times B]\!] \stackrel{\text{def}}{=} [\![A]\!] \times [\![B]\!] \qquad [\![A + B]\!] \stackrel{\text{def}}{=} [\![A]\!] + [\![B]\!]$$

**Skeletal types**

$$[\![P \times Q]\!] \stackrel{\text{def}}{=} [\![P]\!] \times [\![Q]\!] \qquad [\![P \to Q!]\!] \stackrel{\text{def}}{=} [\![P]\!] \Rightarrow [\![Q!]\!]$$

$$[\![P + Q]\!] \stackrel{\text{def}}{=} [\![P]\!] + [\![Q]\!] \qquad [\![P \to Q \not\downarrow C]\!] \stackrel{\text{def}}{=} [\![P]\!] \Rightarrow [\![Q \not\downarrow C]\!]$$

$$[\![\mathsf{runner}\, C]\!] \stackrel{\text{def}}{=} \mathsf{Runner}^{\mathsf{s}}\, [\![C]\!] \qquad [\![P!]\!] \stackrel{\text{def}}{=} \mathsf{U}^{\mathsf{s}}[\![P]\!] \qquad [\![P \not\downarrow C]\!] \stackrel{\text{def}}{=} \mathsf{K}^{\mathsf{s}}_{[\![C]\!]}[\![P]\!]$$

$$[\![x_1 : P_1, \ldots, x_n : P_n]\!] \stackrel{\text{def}}{=} [\![P_1]\!] \times \cdots \times [\![P_n]\!]$$

**Fig. 4.** Denotations of ground and skeletal types.

Semantics of effectful types is given in Fig. 5. From a category-theoretic viewpoint, it assigns meaning in the category Sub(Set) whose objects are subset inclusions $X_0 \subseteq X_1$ and morphisms from $X_0 \subseteq X_1$ to $Y_0 \subseteq Y_1$ those maps $X_1 \to Y_1$ that restrict to $X_0 \to Y_0$. The interpretations of products, sums, and function types are precisely the corresponding category-theoretic notions $\times$, $+$, and $\Rrightarrow$ in Sub(Set). Even better, the pairs of submonads $\mathsf{U}_{\Sigma,E} \subseteq \mathsf{U}^{\mathsf{s}}$ and $\mathsf{K}_{\Sigma,E,S,C} \subseteq \mathsf{K}^{\mathsf{s}}_C$ are the "Sub(Set)-variants" of the user and kernel monads. Such an abstract point of view drives the interpretation of terms, given below, and it additionally suggests how our semantics can be set up on top of a category other than Set. For example, if we replace Set with the category Cpo of $\omega$-complete partial orders, we obtain the domain-theoretic semantics of effect handlers from [3] that models recursion and operations whose signatures contain arbitrary types.

#### 5.2 Semantics of values and computations

To give semantics to $\lambda_{\mathsf{coop}}$'s terms, we introduce *skeletal typing* judgements

$$\Gamma \vdash^{\mathsf{s}} V : P, \qquad \Gamma \vdash^{\mathsf{s}} M : P!, \qquad \Gamma \vdash^{\mathsf{s}} K : P \not\downarrow C,$$

which assign skeletal types to values and computations. In these judgements, $\Gamma$ is a *skeletal context* which assigns skeletal types to variables.

The rules for these judgements are obtained from $\lambda_{\mathsf{coop}}$'s typing rules, by *excluding* subsumption rules and by relaxing restrictions on effects. For example, the skeletal versions of the rules TyValue-Runner and TyKernel-Kill are

$$\frac{\big(\Gamma, x : A_{\mathsf{op}} \vdash^{\mathsf{s}} K_{\mathsf{op}} : B_{\mathsf{op}} \not\downarrow C\big)_{\mathsf{op} \in \Sigma}}{\Gamma \vdash^{\mathsf{s}} \{(\mathsf{op}\, x \mapsto K_{\mathsf{op}})_{\mathsf{op} \in \Sigma}\}_C : \mathsf{runner}\, C} \qquad \frac{s \in \mathcal{S}}{\Gamma \vdash^{\mathsf{s}} \mathsf{kill}_{X @ C}\, s : X^{\mathsf{s}} \not\downarrow C}$$

The relationship between effectful and skeletal typing is summarised as follows:

**Proposition 5.** *(1) Skeletal typing derivations are unique. (2) If $X \sqsubseteq Y$, then $X^{\mathsf{s}} = Y^{\mathsf{s}}$, and analogously for subtyping of user and kernel types. (3) If $\Gamma \vdash V : X$, then $\Gamma^{\mathsf{s}} \vdash^{\mathsf{s}} V : X^{\mathsf{s}}$, and analogously for user and kernel computations.*

**Skeletons**

$$A^{\mathsf{s}} \stackrel{\text{def}}{=} A \qquad (\Sigma \Rightarrow (\Sigma', S, C))^{\mathsf{s}} \stackrel{\text{def}}{=} \mathsf{runner}\, C \qquad (X \times Y)^{\mathsf{s}} \stackrel{\text{def}}{=} X^{\mathsf{s}} \times Y^{\mathsf{s}}$$

$$(X \to Y \,!\, \mathcal{U})^{\mathsf{s}} \stackrel{\text{def}}{=} X^{\mathsf{s}} \to (Y \,!\, \mathcal{U})^{\mathsf{s}} \qquad (X + Y)^{\mathsf{s}} \stackrel{\text{def}}{=} X^{\mathsf{s}} + Y^{\mathsf{s}}$$

$$(X \to Y \,\lightning\, \mathcal{K})^{\mathsf{s}} \stackrel{\text{def}}{=} X^{\mathsf{s}} \to (Y \,\lightning\, \mathcal{K})^{\mathsf{s}} \qquad (X \,!\, \mathcal{U})^{\mathsf{s}} \stackrel{\text{def}}{=} X^{\mathsf{s}}!$$

$$(x_1 : X_1, \ldots, x_n : X_n)^{\mathsf{s}} \stackrel{\text{def}}{=} (x_1 : X_1^{\mathsf{s}}, \ldots, x_n : X_n^{\mathsf{s}}) \qquad (X \,\lightning\, (\Sigma, E, S, C))^{\mathsf{s}} \stackrel{\text{def}}{=} X^{\mathsf{s}} \,\lightning\, C$$

**Denotations**

$$[\![\![ A ]\!]\!] \stackrel{\text{def}}{=} [\![ A ]\!] \qquad [\![\![ X \times Y ]\!]\!] \stackrel{\text{def}}{=} [\![\![ X ]\!]\!] \times [\![\![ X ]\!]\!]$$

$$[\![\![ \Sigma \Rightarrow (\Sigma', S, C) ]\!]\!] \stackrel{\text{def}}{=} \mathsf{Runner}_{\Sigma, \Sigma', S}\, [\![ C ]\!] \qquad [\![\![ X + Y ]\!]\!] \stackrel{\text{def}}{=} [\![\![ X ]\!]\!] + [\![\![ X ]\!]\!]$$

$$[\![\![ X \to Y \,!\, \mathcal{U} ]\!]\!] \stackrel{\text{def}}{=} ([\![\![ X ]\!]\!], [\![ X^{\mathsf{s}} ]\!]) \Rrightarrow ([\![\![ Y \,!\, \mathcal{U} ]\!]\!], [\![ (Y \,!\, \mathcal{U})^{\mathsf{s}} ]\!])$$

$$[\![\![ X \to Y \,\lightning\, \mathcal{K} ]\!]\!] \stackrel{\text{def}}{=} ([\![\![ X ]\!]\!], [\![ X^{\mathsf{s}} ]\!]) \Rrightarrow ([\![\![ Y \,\lightning\, \mathcal{K} ]\!]\!], [\![ (Y \,\lightning\, \mathcal{K})^{\mathsf{s}} ]\!])$$

$$[\![\![ X \,!\, (\Sigma, E) ]\!]\!] \stackrel{\text{def}}{=} \mathsf{U}_{\Sigma, E} [\![\![ X ]\!]\!] \qquad [\![\![ X \,\lightning\, (\Sigma, E, S, C) ]\!]\!] \stackrel{\text{def}}{=} \mathsf{K}_{\Sigma, E, S, [\![ C ]\!]} [\![\![ X ]\!]\!]$$

$$[\![\![ x_1 : X_1, \ldots, x_n : X_n ]\!]\!] \stackrel{\text{def}}{=} [\![\![ X_1 ]\!]\!] \times \cdots \times [\![\![ X_n ]\!]\!]$$

**Fig. 5.** Skeletons and denotations of types.

*Proof.* We prove (1) by induction on skeletal typing derivations, and (2) by induction on subtyping derivations. For (1), we further use the occasional type annotations, and the absence of skeletal subsumption rules. For proving (3), suppose that $\mathcal{D}$ is a derivation of $\Gamma \vdash V : X$. We may translate $\mathcal{D}$ to its *skeleton* $\mathcal{D}^{\mathsf{s}}$ deriving $\Gamma^{\mathsf{s}} \vdash^{\mathsf{s}} V : X^{\mathsf{s}}$ by replacing typing rules with matching skeletal ones, skipping subsumption rules due to (2). Computations are treated similarly. □

To ensure semantic coherence, we first define the *skeletal semantics* of skeletal typing judgements, $[\![ \Gamma \vdash^{\mathsf{s}} V : P ]\!] : [\![ \Gamma ]\!] \to [\![ P ]\!]$, $[\![ \Gamma \vdash^{\mathsf{s}} M : P! ]\!] : [\![ \Gamma ]\!] \to [\![ P! ]\!]$, and $[\![ \Gamma \vdash^{\mathsf{s}} K : P \lightning C ]\!] : [\![ \Gamma ]\!] \to [\![ P \lightning C ]\!]$, by induction on their (unique) derivations.

Provided maps $[\![ A_1 ]\!] \times \cdots \times [\![ A_n ]\!] \to [\![ B ]\!]$ denoting ground constants $\mathsf{f}$, values are interpreted in a standard way, using the bi-cartesian closed structure of sets, except for a runner $\{(\mathsf{op}\, x \mapsto K_{\mathsf{op}})_{\mathsf{op} \in \Sigma}\}_C$, which is interpreted at an environment $\gamma \in [\![ \Gamma ]\!]$ as the skeletal runner $\{\overline{\mathsf{op}} : [\![ A_{\mathsf{op}} ]\!] \to \mathsf{K}_{\mathcal{O}, E_{\mathsf{op}}, \mathcal{S}, [\![ C ]\!]} [\![ B_{\mathsf{op}} ]\!]\}_{\mathsf{op} \in \mathcal{O}}$, given by

$$\overline{\mathsf{op}}\, a \stackrel{\text{def}}{=} (\mathsf{if}\ \mathsf{op} \in \Sigma\ \mathsf{then}\ \rho([\![ \Gamma, x : A_{\mathsf{op}} \vdash^{\mathsf{s}} K_{\mathsf{op}} : B_{\mathsf{op}} \,\lightning\, C ]\!](\gamma, a))\ \mathsf{else}\ \text{⸸}).$$

Here the map $\rho : \mathsf{K}^{\mathsf{s}}_{[\![ C ]\!]} [\![ B_{\mathsf{op}} ]\!] \to \mathsf{K}_{\mathcal{O}, E_{\mathsf{op}}, \mathcal{S}, [\![ C ]\!]} [\![ B_{\mathsf{op}} ]\!]$ is the skeletal kernel theory homomorphism characterised by the equations

$$\rho(\mathsf{return}\ b) = \mathsf{return}\ b, \qquad \rho(\mathsf{op}'(a', \kappa, (\nu_e)_{e \in E_{\mathsf{op}'}})) = \mathsf{op}'(a', \rho \circ \kappa, (\rho(\nu_e))_{e \in E_{\mathsf{op}'}}),$$

$$\rho(\mathsf{getenv}\, \kappa) = \mathsf{getenv}(\rho \circ \kappa), \qquad \rho(\mathsf{raise}\, e) = (\mathsf{if}\ e \in E_{\mathsf{op}}\ \mathsf{then}\ \mathsf{raise}\, e\ \mathsf{else}\ \text{⸸}),$$

$$\rho(\mathsf{setenv}(c, \kappa)) = \mathsf{getenv}(c, \rho \circ \kappa), \qquad \rho(\mathsf{kill}\, s) = \mathsf{kill}\, s.$$

The purpose of ⸸ in the definition of $\overline{\mathsf{op}}$ is to model a runtime error when the runner is asked to handle an unexpected operation, while $\rho$ makes sure that $\overline{\mathsf{op}}$ raises at most the exceptions $E_{\mathsf{op}}$, as prescribed by the signature of $\mathsf{op}$.

User and kernel computations are interpreted as elements of the corresponding skeletal user and kernel monads. Again, most constructs are interpreted in a standard way: returns as the units of the monads; the operations raise, kill, getenv, setenv, and ops as the corresponding algebraic operations; and match statements as the corresponding semantic elimination forms. The interpretation of exception handling offers no surprises, e.g., as in [30], as long as we follow the strategy of treating unexpected situations with the runtime error $\lightning$.

The most interesting part of the interpretation is the semantics of

$$\Gamma \vdash^{\mathsf{s}} (\mathsf{using}\ V \mathbin{@} W\ \mathsf{run}\ M\ \mathsf{finally}\ F) : Q!, \tag{4}$$

where $F \stackrel{\text{def}}{=} \{\mathsf{return}\ x \mathbin{@} c \mapsto N, (\mathsf{raise}\ e \mathbin{@} c \mapsto N_e)_{e \in E}, (\mathsf{kill}\ s \mapsto N_s)_{s \in S}\}$. At an environment $\gamma \in [\![\Gamma]\!]$, $V$ is interpreted as a skeletal runner with state $[\![C]\!]$, which induces a monad morphism $\mathsf{r} : \mathrm{Tree}_{\mathcal{O}}(-) \to ([\![C]\!] \Rightarrow \mathrm{Tree}_{\mathcal{O}}(- \times [\![C]\!] + \mathcal{S}))$, as in the proof of Prop. 3. Let $f : \mathsf{K}^{\mathsf{s}}_{[\![C]\!]}[\![P]\!] \to ([\![C]\!] \Rightarrow \mathsf{U}^{\mathsf{s}}[\![Q]\!])$ be the skeletal kernel theory homomorphism characterised by the equations

$$\begin{aligned} f(\mathsf{return}\, p) &= \lambda c \,.\, [\![\Gamma, x{:}P, c{:}C \vdash^{\mathsf{s}} N : Q]\!](\gamma, p, c), \\ f(\mathsf{op}(a, \kappa, (\nu_e)_{e \in E_{\mathsf{op}}})) &= \lambda c \,.\, \mathsf{op}(a, \lambda b \,.\, f(\kappa\, b)\, c, (f(\nu_e)\, c)_{e \in E_{\mathsf{op}}}), \\ f(\mathsf{raise}\, e) &= \lambda c \,.\, (\mathsf{if}\ e \in E\ \mathsf{then}\ [\![\Gamma, c : C \vdash^{\mathsf{s}} N_e : Q]\!](\gamma, c)\ \mathsf{else}\ \lightning), \\ f(\mathsf{kill}\, s) &= \lambda c \,.\, (\mathsf{if}\ s \in S\ \mathsf{then}\ [\![\Gamma \vdash^{\mathsf{s}} N_s : Q]\!]\, \gamma\ \mathsf{else}\ \lightning), \\ f(\mathsf{getenv}\, \kappa) = \lambda c \,.\, f(\kappa\, c)\, c, & \qquad f(\mathsf{setenv}(c', \kappa)) = \lambda c \,.\, f\, \kappa\, c'. \end{aligned} \tag{5}$$

The interpretation of (4) at $\gamma$ is $f(\mathsf{r}_{[\![P]\!]+\mathcal{E}}([\![\Gamma \vdash^{\mathsf{s}} M : P!]\!]\, \gamma))\, ([\![\Gamma \vdash^{\mathsf{s}} W : C]\!]\, \gamma)$, which reads: map the interpretation of $M$ at $\gamma$ from the skeletal user monad to the skeletal kernel monad using $\mathsf{r}$ (which models the operations of $M$ by the cooperations of $V$), and from there using $f$ to a map $[\![C]\!] \Rightarrow \mathsf{U}^{\mathsf{s}}[\![Q]\!]$, that is then applied to the initial kernel state, namely, the interpretation of $W$ at $\gamma$.

We interpret the context switch $\Gamma \vdash^{\mathsf{s}} \mathsf{kernel}\ K \mathbin{@} W\ \mathsf{finally}\ F : Q!$ at an environment $\gamma \in [\![\Gamma]\!]$ as $f([\![\Gamma \vdash^{\mathsf{s}} K : P \lightning C]\!]\, \gamma)\, ([\![\Gamma \vdash^{\mathsf{s}} W : C]\!]\, \gamma)$, where $f$ is the map (5). Finally, user context switch is interpreted much like exception handling.

We now define coherent semantics of $\lambda_{\mathsf{coop}}$'s typing derivations by passing through the skeletal semantics. Given a derivation $\mathcal{D}$ of $\Gamma \vdash V : X$, its skeleton $\mathcal{D}^{\mathsf{s}}$ derives $\Gamma^{\mathsf{s}} \vdash^{\mathsf{s}} V : X^{\mathsf{s}}$. We identify the denotation of $V$ with the skeletal one,

$$[\![\![\Gamma \vdash V : X]\!]\!] \stackrel{\text{def}}{=} [\![\Gamma^{\mathsf{s}} \vdash^{\mathsf{s}} V : X^{\mathsf{s}}]\!] : [\![\Gamma^{\mathsf{s}}]\!] \to [\![X^{\mathsf{s}}]\!].$$

All that remains is to check that $[\![\![\Gamma \vdash V : X]\!]\!]$ restricts to $[\![\![\Gamma]\!]\!] \to [\![\![X]\!]\!]$. This is accomplished by induction on $\mathcal{D}$. The only interesting step is subsumption, which relies on a further observation that $X \sqsubseteq Y$ implies $[\![\![X]\!]\!] \subseteq [\![\![Y]\!]\!]$. Typing derivations for user and kernel computations are treated analogously.

#### 5.3 Coherence, soundness, and finalisation theorems

We are now ready to prove a theorem that guarantees execution of finalisation code. But first, let us record the fact that the semantics is coherent and sound.

**Theorem 6 (Coherence and soundness).** *The denotational semantics of* $\lambda_{\mathsf{coop}}$ *is coherent, and it is sound for the equational theory of* $\lambda_{\mathsf{coop}}$ *from §4.4.*

*Proof.* Coherence is established by construction: any two derivations of the same typing judgement have the same denotation because they are both (the same) restriction of skeletal semantics. For proving soundness, one just needs to unfold the denotations of the left- and right-hand sides of equations from §4.4, and compare them, where some cases rely on suitable substitution lemmas. □

To set the stage for the finalisation theorem, let us consider the computation $\mathsf{using}\ V \mathbin{@} W\ \mathsf{run}\ M\ \mathsf{finally}\ F$, well-typed by the rule TyUser-Run from Fig. 3. At an environment $\gamma \in [\![\![\Gamma]\!]\!]$, the finalisation clauses $F$ are captured semantically by the *finalisation map* $\phi_\gamma : ([\![\![X]\!]\!] + E) \times [\![\![C]\!]\!] + S \to [\![\![Y \,!\, (\Sigma', E')]\!]\!]$, given by

$$\begin{aligned}\phi_\gamma(\iota_1(\iota_1\, x, c)) &\stackrel{\text{def}}{=} [\![\![\Gamma, x\!:\!X, c\!:\!C \vdash N : Y \,!\, (\Sigma', E')]\!]\!](\gamma, x, c),\\ \phi_\gamma(\iota_1(\iota_2\, e, c)) &\stackrel{\text{def}}{=} [\![\![\Gamma, c\!:\!C \vdash N_e : Y \,!\, (\Sigma', E')]\!]\!](\gamma, c),\\ \phi_\gamma(\iota_2(s)) &\stackrel{\text{def}}{=} [\![\![\Gamma \vdash N_s : Y \,!\, (\Sigma', E')]\!]\!]\,\gamma.\end{aligned}$$

With $\phi$ in hand, we may formulate the finalisation theorem for $\lambda_{\mathsf{coop}}$, stating that the semantics of $\mathsf{using}\ V \mathbin{@} W\ \mathsf{run}\ M\ \mathsf{finally}\ F$ is a computation tree all of whose branches end with finalisation clauses from $F$. Thus, unless some enveloping runner sends a signal, finalisation with $F$ is guaranteed to take place.

**Theorem 7 (Finalisation).** *A well-typed* $\mathsf{run}$ *factors through finalisation:*

$$[\![\![\Gamma \vdash (\mathsf{using}\ V \mathbin{@} W\ \mathsf{run}\ M\ \mathsf{finally}\ F) : Y \,!\, (\Sigma', E')]\!]\!]\,\gamma = \phi^\dagger_\gamma\, t,$$

*for some* $t \in \mathrm{Tree}_{\Sigma'}\,(([\![\![X]\!]\!] + E) \times [\![\![C]\!]\!] + S)$.

*Proof.* We first prove that $f\,u\,c = \phi^\dagger_\gamma(u\,c)$ holds for all $u \in \mathsf{K}_{\Sigma', E, S, [\![\![C]\!]\!]}[\![\![X]\!]\!]$ and $c \in [\![\![C]\!]\!]$, where $f$ is the map (5). The proof proceeds by computational induction on $u$ [29]. The finalisation statement is then just the special case with $u \stackrel{\text{def}}{=} \mathsf{r}_{[\![\![X]\!]\!]+E}([\![\![\Gamma \vdash M : X \,!\, (\Sigma, E)]\!]\!]\,\gamma)$ and $c \stackrel{\text{def}}{=} [\![\![\Gamma \vdash W : C]\!]\!]\,\gamma$. □

### 6 Runners in action

Let us show examples that demonstrate how runners can be usefully combined to provide flexible resource management. We implemented these and other examples in the language Coop and a library Haskell-Coop, see §7.

To make the code more understandable, we do not adhere strictly to the syntax of $\lambda_{\mathsf{coop}}$, e.g., we use the generic versions of effects [26], as is customary in programming, and effectful initialisation of kernel state as discussed in §3.2.

*Example 8 (Nesting).* In Example 4, we considered a runner fileIO for basic file operations. Let us suppose that fileIO is implemented by immediate calls to the operating system. Sometimes, we might prefer to accumulate writes and commit them all at once, which can be accomplished by interposing between fileIO and user code the following runner accIO, which accumulates writes in its state:

```
{ write s' → let s = getenv () in setenv (concat s s') }string
```

By *nesting* the runners, and calling the outer write (the one of fileIO) only in the finalisation code for accIO, the accumulated writes are commited all at once:

```
using fileIO @ (open "hello.txt") run
  using accIO @ (return "") run
    write "Hello, world."; write "Hello, again."
  finally { return x @ s → write s; return x }
finally { return x @ fh → ... , raise QuotaExceeded @ fh → ... , kill IOError → ... }
```

*Example 9 (Instrumentation).* Above, accIO implements the same signature as fileIO and thus intercepts operations without the user code being aware of it. This kind of invisibility can be more generally used to implement *instrumentation*:

```
using { ..., op x → let c = getenv () in setenv (c+1); op x, ... }int @ (return 0) run
  M
finally { return x @ c → report_cost c; return x, ... }
```

Here the interposed runner implements all operations of some enveloping runner, by simply forwarding them, while also measuring computational cost by counting the total number of operation calls, which is then reported during finalisation.

*Example 10 (ML-style references).* Continuing with the theme of nested runners, they can also be used to implement abstract and safe interfaces to low-level resources. For instance, suppose we have a low-level implementation of a memory heap that potentially allows unsafe memory access, and we would like to implement ML-style references on top of it. A good first attempt is the runner

```
{ ref x → let h = getenv () in
          let (r,h') = malloc h x in
          setenv h'; return r,
  get r → let h = getenv () in memread h r,
  put (r, x) → let h = getenv () in memset h r x }heap
```

which has the desired interface, but still suffers from three deficiencies that can be addressed with further language support. First, *abstract types* would let us hide the fact that references are just memory locations, so that the user code could never devise invalid references or otherwise misuse them. Second, our simple typing discipline forces all references to hold the same type, but in reality we want them to have different types. This could be achieved through quantification over types in the low-level implementation of the heap, as we have done in the HASKELL-COOP library using HASKELL's forall. Third, user code could hijack a reference and misuse it out of the scope of the runner, which is difficult to prevent. In practice the problem does not occur because, so to speak, the runner for references is at the very top level, from which user code cannot escape.

*Example 11 (Monotonic state).* Nested runners can also implement access restrictions to resources, with applications in security [8]. For example, we can restrict the references from the previous example to be used *monotonically* by associating a preorder with each reference, which assignments then have to obey. This idea is similar to how monotonic state is implemented in the F* language [2], except that we make dynamic checks where F* statically uses dependent types.

While we could simply modify the previous example, it is better to implement a new runner which is nested inside the previous one, so that we obtain a modular solution that works with *any* runner implementing operations ref, get, and put:

```
{ mref x rel → let r = ref x in
               let m = getenv () in
               setenv (add m (r,rel)); return r,
  mget r → get r,
  mput (r, y) → let x = get r in
                let m = getenv () in
                match (sel m r) with
                | inl rel → if (rel x y) then put (r, y)
                                         else raise MonotonicityViolation
                | inr () → kill NoPreoderFound }map(ref,intRel)
```

The runner's state is a map from references to preorders on integers. The co-operation mref x rel creates a new reference r initialised with x (by calling ref of the outer runner), and then adds the pair (r, rel) to the map stored in the runner's state. Reading is delegated to the outer runner, while assignment first checks that the new state is larger than the old one, according to the associated preorder. If the preorder is respected, the runner proceeds with assignment (again delegated to the outer runner), otherwise it reports a monotonicity violation. We may not assume that every reference has an associated preorder, because user code could pass to mput a reference that was created earlier outside the scope of the runner. If this happens, the runner simply kills the offending user code with a signal.

*Example 12 (Pairing).* Another form of modularity is achieved by *pairing* runners. Given two runners $\{(\mathsf{op}\, x \mapsto K_{\mathsf{op}})_{\mathsf{op} \in \Sigma_1}\}_{C_1}$ and $\{(\mathsf{op}'\, x \mapsto K_{\mathsf{op}'})_{\mathsf{op}' \in \Sigma_2}\}_{C_2}$, e.g., for state and file operations, we can use them side-by-side by combining them into a single runner with operations $\Sigma_1 + \Sigma_2$ and kernel state $C_1 \times C_2$, as follows (the co-operations $\mathsf{op}'$ of the second runner are treated symmetrically):

```
{ op x → let (c,c') = getenv () in
         user
           kernel (K_op x) @ c finally {
              return y @ c'' → return (inl (inl y, c'')),
              (raise e @ c'' → return (inl (inr e, c'')))e∈E_op,
              (kill s → return (inr s))s∈S1}
         with {
           return (inl (inl y, c'')) → setenv (c'', c'); return y,
           return (inl (inr e, c'')) → setenv (c'', c'); raise e,
           return (inr s) → kill s},
  op' x → ... , ... }C1×C2
```

Notice how the inner kernel context switch passes to the co-operation $K_{\mathsf{op}}$ only its part of the combined state, and how it returns the result of $K_{\mathsf{op}}$ in a reified form (which requires treating exceptions and signals as values). The outer user context switch then receives this reified result, updates the combined state, and forwards the result (return value, exception, or signal) in unreified form.

### 7 Implementation

We accompany the theoretical development with two implementations of $\lambda_{\mathsf{coop}}$: a prototype language Coop [6], and a Haskell library Haskell-Coop [1].

Coop, implemented in OCaml, demonstrates what a more fully-featured language based on $\lambda_{\mathsf{coop}}$ might look like. It implements a bi-directional variant of $\lambda_{\mathsf{coop}}$'s type system, extended with type definitions and algebraic datatypes, to provide algorithmic typechecking and type inference. The operational semantics is based on the computation rules of the equational theory from §4.4, but extended with general recursion, pairing of runners from Example 12, and an interface to the OCaml runtime called *containers*—these are essentially top-level runners defined directly in OCaml. They are a modular and systematic way of offering several possible top-level runtime environments to the programmer.

The Haskell-Coop library is a shallow embedding of $\lambda_{\mathsf{coop}}$ in Haskell. The implementation closely follows the denotational semantics of $\lambda_{\mathsf{coop}}$. For instance, user and kernel monads are implemented as corresponding Haskell monads. Internally, the library uses the Freer monad of Kiselyov [14] to implement free model monads for given signatures of operations. The library also provides a means to run user code via Haskell's top-level monads. For instance, code that performs input-output operations may be run in Haskell's IO monad.

Haskell's advanced features make it possible to use Haskell-Coop to implement several extensions to examples from §6. For instance, we implement ML-style state that allow references holding arbitrary values (of different types), and state that uses Haskell's type system to track which references are alive. The library also provides pairing of runners from Example 12, e.g., to combine state and input-output. We also use the library to demonstrate that *ambient functions* from the Koka language [18] can be implemented with runners by treating their binding and application as co-operations. (These are functions that are bound dynamically but evaluated in the lexical scope of their binding.)

### 8 Related work

Comodels and (ordinary) runners have been used as a natural model of stateful top-level behaviour. For instance, Plotkin and Power [27] have given a treatment of operational semantics using the tensor product of a model and a comodel. Recently, Katsumata, Rivas, and Uustalu have generalised this interaction of models and comodels to monads and comonads [13]. An early version of Eff [4] implemented *resources*, which were a kind of stateful runners, although they lacked satisfactory theory. Uustalu [35] has pointed out that runners are the additional structure that one has to impose on state to run algebraic effects statefully. Møgelberg and Staton's [21] linear-use state-passing translation also relies on equipping the state with a comodel structure for the effects at hand. Our runners arise when their setup is specialised to a certain Kleisli adjunction.

Our use of kernel state is analogous to the use of parameters in parameter-passing handlers [30]: their return clause also provides a form of finalisation, as the final value of the parameter is available. There is however no guarantee of finalisation happening because handlers need not use the continuation linearly.

The need to tame the excessive generality of handlers, and willingness to give it up in exchange for efficiency and predictability, has recently been recognised by Multicore OCaml's implementors, who have observed that in practice most handlers resume continuations precisely once [9]. In exchange for impressive efficiency, they require continuations to be used linearly by default, whereas discarding and copying must be done explicitly, incurring additional cost. Leijen [17] has extended handlers in Koka with a finally clause, whose semantics ensures that finalisation happens whenever a handler discards its continuation. Leijen also added an initially clause to parameter-passing handlers, which is used to compute the initial value of the parameter before handling, but that gets executed again every time the handler resumes its continuation.

### 9 Conclusion and future work

We have shown that effectful runners form a mathematically natural and modular model of resources, modelling not only the top level external resources, but allowing programmers to also define their own intermediate "virtual machines". Effectful runners give rise to a bona fide programming concept, an idea we have captured in a small calculus, called $\lambda_{\mathsf{coop}}$, which we have implemented both as a language and a library. We have given $\lambda_{\mathsf{coop}}$ an algebraically natural denotational semantics, and shown how to program with runners through various examples.

We leave combining runners and general effect handlers for future work. As runners are essentially affine handlers, inspired by Multicore OCaml we also plan to investigate efficient compilation for runners. On the theoretical side, by developing semantics in a Sub(Cpo)-enriched setting [32], we plan to support recursion at all levels, and remove the distinction between ground and arbitrary types. Finally, by using proof-relevant subtyping [34] and synthesis of lenses [20], we plan to upgrade subtyping from a simple inclusion to relating types by lenses.

*Acknowledgements* We thank Daan Leijen for useful discussions about initialisation and finalisation in Koka, as well as ambient values and ambient functions. We thank Guillaume Munch-Maccagnoni and Matija Pretnar for discussing resources and potential future directions for $\lambda_{\mathsf{coop}}$. We are also grateful to the participants of the NII Shonan Meeting "Programming and reasoning with algebraic effects and effect handlers" for feedback on an early version of this work.

This project has received funding from the European Union's Horizon 2020 research and innovation programme under the Marie Skłodowska-Curie grant agreement No 834146.

This material is based upon work supported by the Air Force Office of Scientific Research under award number FA9550-17-1-0326.

## References

[1] Ahman, D.: Library Haskell-Coop. Available at https://github.com/danelahman/haskell-coop (2019)

[2] Ahman, D., Fournet, C., Hritcu, C., Maillard, K., Rastogi, A., Swamy, N.: Recalling a witness: foundations and applications of monotonic state. PACMPL **2**(POPL), 65:1–65:30 (2018)

[3] Bauer, A., Pretnar, M.: An effect system for algebraic effects and handlers. Logical Methods in Computer Science **10**(4) (2014)

[4] Bauer, A., Pretnar, M.: Programming with algebraic effects and handlers. J. Log. Algebr. Meth. Program. **84**(1), 108–123 (2015)

[5] Bauer, A.: What is algebraic about algebraic effects and handlers? CoRR **abs/1807.05923** (2018)

[6] Bauer, A.: Programming language coop. Available at https://github.com/andrejbauer/coop (2019)

[7] Benton, N., Kennedy, A.: Exceptional syntax. Journal of Functional Programming **11**(4), 395–410 (2001)

[8] Delignat-Lavaud, A., Fournet, C., Kohlweiss, M., Protzenko, J., Rastogi, A., Swamy, N., Zanella-Beguelin, S., Bhargavan, K., Pan, J., Zinzindohoue, J.K.: Implementing and proving the tls 1.3 record layer. In: 2017 IEEE Symp. on Security and Privacy (SP). pp. 463–482 (2017)

[9] Dolan, S., Eliopoulos, S., Hillerström, D., Madhavapeddy, A., Sivaramakrishnan, K.C., White, L.: Concurrent system programming with effect handlers. In: Wang, M., Owens, S. (eds.) Trends in Functional Programming. pp. 98–117. Springer International Publishing, Cham (2018)

[10] Foster, J.N., Greenwald, M.B., Moore, J.T., Pierce, B.C., Schmitt, A.: Combinators for bidirectional tree transformations: A linguistic approach to the view-update problem. ACM Trans. Program. Lang. Syst. **29**(3) (2007)

[11] Hyland, M., Plotkin, G., Power, J.: Combining effects: Sum and tensor. Theor. Comput. Sci. **357**(1–3), 70–99 (2006)

[12] Kammar, O., Lindley, S., Oury, N.: Handlers in action. In: Proc. of 18th ACM SIGPLAN Int. Conf. on Functional Programming, ICFP 2013. ACM (2013)

[13] Katsumata, S., Rivas, E., Uustalu, T.: Interaction laws of monads and comonads. CoRR **abs/1912.13477** (2019)

[14] Kiselyov, O., Ishii, H.: Freer monads, more extensible effects. In: Proc. of 2015 ACM SIGPLAN Symp. on Haskell. pp. 94–105. Haskell ’15, ACM (2015)

[15] Koopman, P., Fokker, J., Smetsers, S., van Eekelen, M., Plasmeijer, R.: Functional Programming in Clean. University of Nijmegen (1998), draft

[16] Leijen, D.: Structured asynchrony with algebraic effects. In: Proceedings of the 2nd ACM SIGPLAN International Workshop on Type-Driven Development, TyDe@ICFP 2017, Oxford, UK, September 3, 2017. pp. 16–29. ACM (2017)

[17] Leijen, D.: Algebraic effect handlers with resources and deep finalization. Tech. Rep. MSR-TR-2018-10, Microsoft Research (April 2018)

[18] Leijen, D.: Programming with implicit values, functions, and control (or, implicit functions: Dynamic binding with lexical scoping). Tech. Rep. MSR-TR-2019-7, Microsoft Research (March 2019)

[19] Levy, P.B.: Call-By-Push-Value: A Functional/Imperative Synthesis, Semantics Structures in Computation, vol. 2. Springer (2004)

[20] Miltner, A., Maina, S., Fisher, K., Pierce, B.C., Walker, D., Zdancewic, S.: Synthesizing symmetric lenses. Proc. ACM Program. Lang. **3**(ICFP), 95:1–95:28 (2019)

[21] Møgelberg, R.E., Staton, S.: Linear usage of state. Logical Methods in Computer Science **10**(1) (2014)

[22] Moggi, E.: Computational lambda-calculus and monads. In: Proc. of 4th Ann. Symp. on Logic in Computer Science, LICS 1989. pp. 14–23. IEEE (1989)

[23] Moggi, E.: Notions of computation and monads. Inf. Comput. **93**(1), 55–92 (1991)

[24] O’Connor, R.: Functor is to lens as applicative is to biplate: Introducing multiplate. CoRR **abs/1103.2841** (2011)

[25] Plotkin, G., Power, J.: Semantics for algebraic operations. In: Proc. of 17th Conf. on the Mathematical Foundations of Programming Semantics, MFPS XVII. ENTCS, vol. 45, pp. 332–345. Elsevier (2001)

[26] Plotkin, G., Power, J.: Algebraic operations and generic effects. Appl. Categor. Struct. (1), 69–94 (2003)

[27] Plotkin, G., Power, J.: Tensors of comodels and models for operational semantics. In: Proc. of 24th Conf. on Mathematical Foundations of Programming Semantics, MFPS XXIV. ENTCS, vol. 218, pp. 295–311. Elsevier (2008)

[28] Plotkin, G.D., Power, J.: Notions of computation determine monads. In: Proc. of 5th Int. Conf. on Foundations of Software Science and Computation Structures, FOSSACS 2002. LNCS, vol. 2303, pp. 342–356. Springer (2002)

[29] Plotkin, G.D., Pretnar, M.: A logic for algebraic effects. In: Proc. of 23th Ann. IEEE Symp. on Logic in Computer Science, LICS 2008. pp. 118–129. IEEE (2008)

[30] Plotkin, G.D., Pretnar, M.: Handling algebraic effects. Logical Methods in Computer Science **9**(4:23) (2013)

[31] Power, J., Shkaravska, O.: From comodels to coalgebras: State and arrays. Electr. Notes Theor. Comput. Sci. **106**, 297–314 (2004)

[32] Power, J.: Enriched Lawvere theories. Theory Appl. Categ **6**(7), 83–93 (1999)

[33] Pretnar, M.: The Logic and Handling of Algebraic Effects. Ph.D. thesis, School of Informatics, University of Edinburgh (2010)

[34] Saleh, A.H., Karachalias, G., Pretnar, M., Schrijvers, T.: Explicit effect subtyping. In: Proc. of 27th European Symposium on Programming, ESOP 2018. pp. 327–354. LNCS, Springer (2018)

[35] Uustalu, T.: Stateful runners of effectful computations. Electr. Notes Theor. Comput. Sci. **319**, 403–421 (2015)

[36] Wadler, P.: The essence of functional programming. In: Sethi, R. (ed.) Proc. of 19th Ann. ACM SIGPLAN-SIGACT Symp. on Principles of Programming Languages, POPL 1992. pp. 1–14. ACM (1992)

